# Structure of an antagonist-bound ghrelin receptor reveals possible ghrelin recognition mode

**DOI:** 10.1038/s41467-020-17554-1

**Published:** 2020-08-19

**Authors:** Yuki Shiimura, Shoichiro Horita, Akie Hamamoto, Hidetsugu Asada, Kunio Hirata, Misuzu Tanaka, Kenji Mori, Tomoko Uemura, Takuya Kobayashi, So Iwata, Masayasu Kojima

**Affiliations:** 1 Division of Molecular Genetics, Institute of Life Science, Kurume University, Fukuoka, Japan; 2 Department of Cell Biology, Graduate School of Medicine, Kyoto University, Kyoto, Japan; 3 RIKEN, SPring-8 Center, Hyogo, Japan; 4 Japan Science and Technology Agency (JST), Precursory Research for Embryonic Science and Technology (PRESTO), Saitama, Japan; 5 Department of Biochemistry, National Cerebral and Cardiovascular Center Research Institute, Suita, Osaka Japan

**Keywords:** Peptide hormones, X-ray crystallography

## Abstract

Ghrelin is a gastric peptide hormone with important physiological functions. The unique feature of ghrelin is its Serine 3 acyl-modification, which is essential for ghrelin’s activity. However, it remains to be elucidated why the acyl-modification of ghrelin is necessary for activity. To address these questions, we solved the crystal structure of the ghrelin receptor bound to antagonist. The ligand-binding pocket of the ghrelin receptor is bifurcated by a salt bridge between E124 and R283. A striking feature of the ligand-binding pocket of the ghrelin receptor is a wide gap (crevasse) between the TM6 and TM7 bundles that is rich in hydrophobic amino acids, including a cluster of phenylalanine residues. Mutagenesis analyses suggest that the interaction between the gap structure and the acyl acid moiety of ghrelin may participate in transforming the ghrelin receptor into an active conformation.

## Abstract (web summary)

Ghrelin is a gastric peptide hormone with important physiological functions, including growth hormone release and appetite-stimulating activity. Here, authors solved the crystal structure of the ghrelin receptor bound to antagonist and suggested a possible mechanism of activation by acyl-modified ghrelin.

## Introduction

Ghrelin, a peptide hormone consisting of 28 amino acids, was originally discovered in the stomach as an endogenous ligand for GHSR (growth hormone secretagogue receptor, now called the ghrelin receptor), a member of the β-branch in the class A GPCRs^1,2^. Ghrelin is the only peptide hormone that induces appetite stimulation following peripheral injection, and is thus functionally opposite to leptin^3^. Moreover, ghrelin has a wide range of physiological functions, playing roles in growth hormone secretion, adiposity, energy homeostasis, memory formation, and hippocampal neurogenesis^4^.

A salient feature of ghrelin is the *O*-acyl-modification at Ser3^2,5^, which is essential for its activity; des-acyl ghrelin (i.e., ghrelin lacking the acyl-modification) is inactive. No other peptide hormone is known to require such an acyl-modification for its activity. The acyl-modification of ghrelin is catalyzed by ghrelin *O*-acyltransferase (GOAT)^6–8^. Although GOAT can transfer fatty acids ranging in size from acetic to palmitic acid onto des-acyl ghrelin, GOAT prefers medium-chain fatty acids as its substrates^9^. The main acyl-modified and most potent active form of ghrelin is n-octanoyl ghrelin, but other medium-chain fatty acids are also detected as endogenous forms of ghrelin. For example, n-decanoyl ghrelin has been purified from the stomachs of frog and bird at levels comparable to those of n-octanoyl ghrelin^10,11^. Moreover, calcium mobility assays using synthesized ghrelin with fatty acids of various lengths revealed that ghrelin is less active when modified with a shorter-chain fatty acid^12^. In addition, several reports indicated that the ghrelin receptor exhibits strong ligand-independent activity^13–15^. The structural basis for its high constitutive activity is thought to be an aromatic amino acid cluster on transmembrane helixes 6 and 7^15^. However, because of the lack of structural information about the ghrelin receptor, the precise mechanisms of its high constitutive activity and activation by acyl-modified ghrelin remained to be elucidated.

Here, we report the first high-resolution crystal structure of the ghrelin receptor bound to an antagonist. We found that the ghrelin receptor has a wide gap (crevasse) between the TM6 and TM7 bundles, which is rich in hydrophobic amino acids including a cluster of phenylalanine residues. Thus, the interaction between the gap structure and the acyl acid moiety of ghrelin may participate in transforming the ghrelin receptor into an active conformation.

## Results

### Crystal structure of the ghrelin receptor

**Fig. 1:**
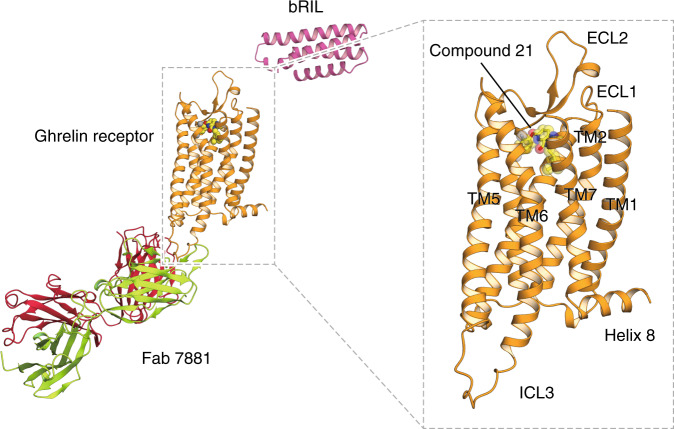
Structure of the antagonist-bound human ghrelin receptor. Overall structure of the bRIL–hGHSR–Fab 7881–Compound 21 complex. The ghrelin receptor is shown in cartoon representation and colored in orange. The bRIL fusion protein is shown in pink cartoon representation. For Fab 7881, the crimson and green-yellow cartoons represent the heavy and light chains, respectively. Compound 21 is shown as spheres and sticks with carbon atoms in yellow, oxygen in red, and nitrogen in blue.

To facilitate the determination of the structure, we designed an engineered ghrelin receptor construct (Supplementary Fig. 1). For crystallization, 28 and 20 residues were removed from the N and C termini of the receptor, respectively, and the thermostabilized apocytochrome b_562_RIL (bRIL) protein from *Escherichia coli* was fused to the deleted N terminus. Deletion of the N-terminal 28 amino acids resulted in loss of cell-surface expression and receptor activity, whereas both expression and activity were retained when the C-terminal 20-amino acids were deleted (Supplementary Fig. 2a–d). In addition, two mutations were introduced: T130^3.39^K (superscripts indicate residue numbering using the Ballesteros–Weinstein nomenclature) to improve thermostability and expression^16^, and N188Q in extracellular loop 2 (ECL2) to avoid glycosylation. Moreover, both a Fab fragment antibody (Fab 7881) specific for the ghrelin receptor and an antagonist, Compound 21, were added to bRIL-conjugated truncated ghrelin receptor to increase thermostability (Supplementary Fig. 3a, b). Compound 21 was synthesized from YIL-781, another ghrelin receptor antagonist, to improve antagonistic activity and lipophilic efficiency^17^. Compound 21 had no effect on the constitutive activity of the ghrelin receptor, indicating that it is a neutral antagonist (Supplementary Fig. 3c). Binding assays using [^3^H]-Compound 21 confirmed that Compound 21 binds to the ghrelin receptor construct, although the binding affinity of the construct (dissociation constant, *K*d = 17.2 ± 7.1 nM) was lower than that of the WT ghrelin receptor (*K*d = 4.42 ± 0.76 nM) (Supplementary Fig. 3d). Fab 7881 binds to intracellular loop 3 (ICL3); the flexibility of ICL3 prevents the crystallization of the receptor. Thus, Fab 7881 seems to stabilize ICL3 and improve the thermostability of the ghrelin receptor, allowing it to form crystals. The ghrelin receptor was crystallized in lipidic cubic phase and solved at 3.3 Å resolution (Fig. 1, Supplementary Fig. 3e–g, Supplementary Table 1).

The ghrelin receptor structure, as a class A GPCR, shares a canonical seven-transmembrane helical architecture and an intracellular amphipathic helix 8 (Fig. 1)^18^. Similar to other peptide hormone receptors, ECL2 forms anti-parallel β-strands with a short hairpin and is stabilized by the highly conserved disulfide bond between C116^3.28^ and C198^ECL2^. ECL3 between TM6 and TM7 has not been observed, probably due to its unstable structure. The structure superimposed well with that of the inactive OX_2_ orexin receptor (PDB ID: 4S0V)^19^, with a root-mean-squared deviation of 0.819 Å. Thus, our structure of the ghrelin receptor probably represents the inactive state.

### Bifurcated ligand-binding pocket and the gap structure

**Fig. 2:**
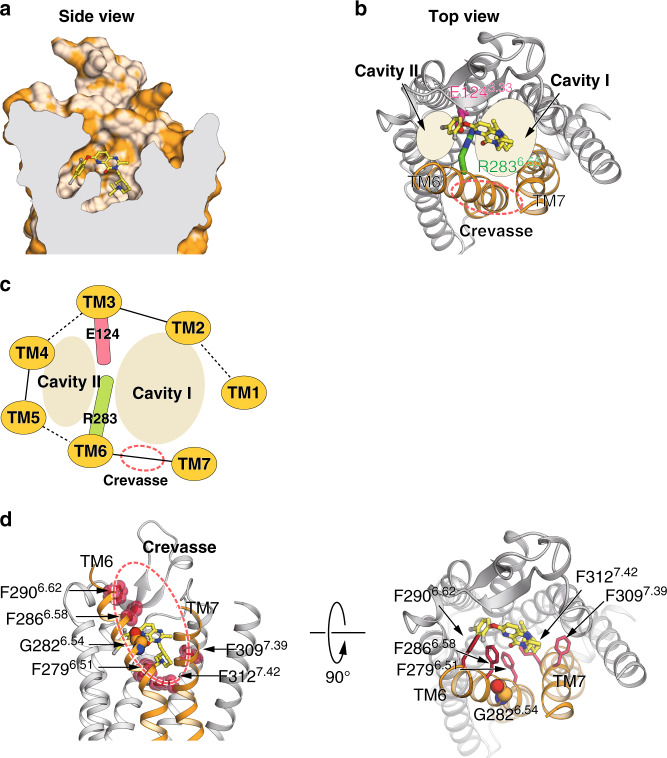
Bifurcated ligand-binding pocket in the ghrelin receptor, and the structure of the gap. **a** The cutaway surface for the ghrelin receptor in complex with the antagonist Compound 21 reveals a bifurcated ligand-binding pocket. **b** The ligand-binding pocket of the ghrelin receptor. **c** Schematic model of Fig. 2b. **d** The phenylalanine cluster forms a crevasse in the ghrelin receptor. The five phenylalanines are shown as sticks in dark orange. The crevasse is shown as a red dashed line. G282 is shown as spheres with carbon atoms in orange, nitrogen in blue, and oxygen in red.

The ghrelin receptor has two main differences relative to other peptide hormone GPCRs. It is the bifurcated ligand-binding pocket and the hydrophobic wide gap structure of TM6 and TM7. The binding pocket of ghrelin receptor is bifurcated into two cavities by a salt bridge between E124^3.33^ and R283^6.55^ (Fig. 2a–c, Supplementary Fig. 4). We call the larger one cavity I, and the smaller one cavity II (Fig. 2b, c). In addition, the top of cavity II is covered with ECL2. Note that the A204E mutation in ECL2 causes loss of constitutive activity of the ghrelin receptor and is associated with familial short stature^20^. In fact, we observed loss of basal receptor activity and a decrease in ghrelin-induced receptor function (Supplementary Fig. 5a, b). Thus, mutation of A204 may block ghrelin’s access to the cavities.

No such bifurcated pocket has been observed in the class A peptide hormone GPCRs in the β-branch family whose structures have been determined thus far (e.g., the neurotensin receptor NTSR1^21^, the OX_1_ and OX_2_ orexin receptors^19,22,23^, the endothelin type B receptor^24^, and the NPY Y_1_ receptor^25^) (Supplementary Fig. 6a–e).

Another characteristic feature of ghrelin receptor is the gap structure between TM6 and TM7. The extracellular side of the TM7 bundle curves outwardly to a greater degree than the corresponding structures of other class A GPCRs. Thus, the TM6 and TM7 bundles kink each other. Furthermore, the G282^6.54^ and the bulky side chains of the five phenylalanines (F279^6.51^, F286^6.58^, F290^6.62^, F309^7.39^, and F312^7.42^) in TM6 and TM7 are positioned to open the space between the TM6 and TM7 bundles. These structural features enable the ghrelin receptor to form a wide gap between the TM6 and TM7 bundles. We refer to this gap as the crevasse (Fig. 2b, d, Supplementary Fig. 7a). However, within the β-branch of class A GPCRs, with the exception of the ghrelin receptor, the TM6–TM7 interface is occupied by bulky side chains that completely separate the ligand binding cavity from the lipid bilayer; consequently, ligands cannot be observed from outside the receptors (Supplementary Fig. 7b–f). In contrast to the peptide hormone GPCRs, several GPCRs with lipid ligands have gaps between TM1 and TM7 (S1P_1_, EP_4_, and TP)^26–28^, TM3 and TM4 (GPR40)^29^, and TM4 and TM5 (PAFR and LPA_6_)^30,31^ (Supplementary Fig. 7g–l). These gaps have been proposed to be the access routes of the lipid ligands from outside the lipid bilayers into the pockets where they bind.

### Binding mode of Compound 21

**Fig. 3:**
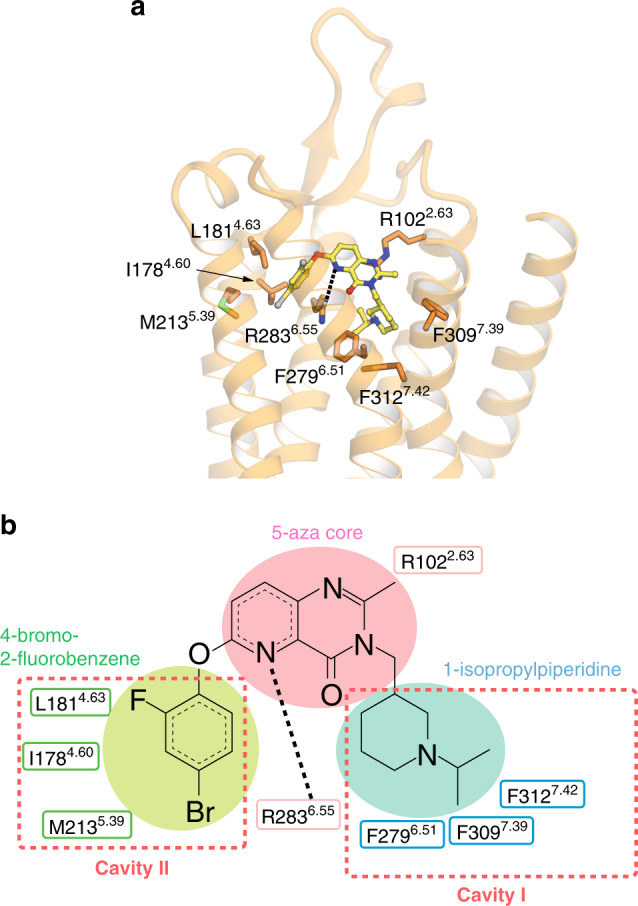
Binding mode of Compound 21. **a** Side chain interactions within 4.0 Å residues are shown in stick representation. Hydrogen bonds are shown as black dashed lines. **b** Schematic representation of the interactions between the ghrelin receptor and Compound 21, analyzed using the Discovery Studio 2016. The black dot line indicates a hydrogen bond.

Compound 21 covers the salt bridge and fills the two cavities (Fig. 3a, b). The crystal structure of ghrelin receptor bound to Compound 21 revealed that the 1-isopropylpiperidine moiety of the Compound 21 sits in cavity I, whereas the 4-bromo-2-fluorobenzene moiety sits in cavity II, and the 5-azaquinazolinone moiety (5-aza core) links between cavities I and II. Moreover, the 5-nitrogen of the 5-aza core forms a hydrogen bond with R283^6.55^, which connects with E124^3.33^ through a salt bridge and forms the boundary between the cavities I and II (Fig. 2b, Supplementary Fig. 4). In addition, the 5-aza core makes a π–cation interaction with R102^2.63^ (Fig. 3a, b). The nitrogen of the 1-isopropylpiperidine moiety of Compound 21 forms hydrophobic interactions with the F279^6.51^, F309^7.39^, and F312^7.42^ (Fig. 3a, b). In particular, the F279^6.51^A and F312^7.42^A mutants decreased the specific binding of [^3^H]-Compound 21 and basal receptor activity (Supplementary Fig. 8f, i, j, Supplementary Fig. 9k), although cell-surface expression of the mutant ghrelin receptors was retained (Supplementary Fig. 9f, j). The 4-bromo-2-fluorobenzene moiety makes three hydrophobic contacts with residues I178^4.60^, L181^4.63^, and M213^5.39^ in cavity II. The fact that Compound 21 covers the two cavities and interacts with eight residues may explain the high antagonistic affinity of Compound 21 for the ghrelin receptor. However, the results of the binding assay using [^3^H]-Compound 21 and site-directed mutants of the ghrelin receptor suggest that the amino acids in cavity I are more important than those in the cavity II for the interaction between Compound 21 and the receptor (Supplementary Fig. 8a–j, Supplementary Fig. 9, Supplementary Fig. 10).

### Amino acid residues in the ligand-binding pocket

**Fig. 4:**
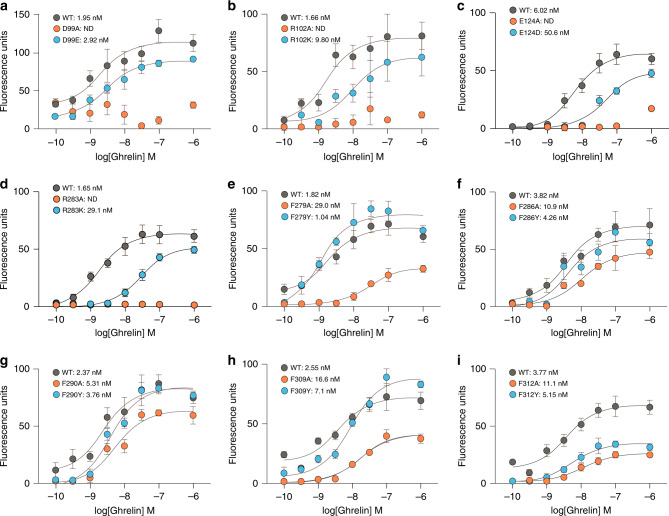
Mutagenesis analyses. Mutagenesis analyses of the polar amino acids of ligand-binding pocket (**a**–**d**) and the five phenylalanines forming the crevasse (**e**–**i**). Receptor activities were examined by intracellular Ca^2+^ concentration assay. EC50 values are means ± s.e.m. (*n* = 4).

Mutations in the salt bridge, E124^3.33^A and R283^6.55^A, completely abolished ghrelin-induced receptor function, whereas replacement with the cognate amino acid mutants E124^3.33^D and R283^6.55^K retained function, although their ghrelin-induced receptor activities were significantly reduced (Fig. 4c, d). Note that the cell-surface expression of E124^3.33^A mutant was retained at 111.5% of the WT level, while that of R283^6.55^A mutant was significantly decreased (66.3% of the WT level) (Supplementary Fig. 9a, d, e). Recent NMR data and structural modeling suggest that the N-terminus of ghrelin extends down into the bottom of the ligand-binding pocket of the receptor, where it interacts with E124^3.33^ ^32^. Furthermore, alanine mutation of two other polar amino acids in the ligand-binding pocket, D99^2.60^ and R102^2.63^, abolishes the receptor activity (Fig. 4a, b). Polar amino acids in the ligand-binding pocket are also important in other peptide hormone GPCRs, such as R327^6.54^ in NTSR1 (PDB ID: 4GRV)^21^, R343^6.55^ and R357^7.24^ in ET_B_ (PDB ID: 5GLH)^24^, and K215^5.42^, D279^6.58^, and D297^7.32^ in AT_2_R (PDB ID: 5XJM)^33^. These polar amino acids interact with the peptide core and side chains to fix the ligand in the binding pocket.

In addition, our crystal structure of the ghrelin receptor suggests a possible mechanism for receptor activation by acyl-modified ghrelin, and in particular the role of the acyl acid. We propose that the interaction between the acyl acid of ghrelin and the hydrophobic environment in the crevasse induces the transformation of the ghrelin receptor into an active form. Mutagenesis analyses of the phenylalanine cluster in the crevasse suggested that the cluster is important for the receptor activity. When F279^6.51^, F309^7.39^, and F312^7.42^ were individually mutated to alanine, basal and ghrelin-induced receptor activities were significantly reduced (Fig. 4e, h, i, Supplementary Fig. 9k), although the cell-surface expression of the mutant receptors was retained at 78.9 − 112.1% of the WT level. By contrast, the basal and ghrelin-induced receptor activities of the F286^6.58^A and F290^6.62^A mutants were only slightly lower than that of the WT ghrelin receptor (Fig. 4f, g, Supplementary Fig. 9k). Moreover, when the phenylalanines were each mutated to tyrosine, the receptor activities were remained at WT levels, with the exception of F312^7.42^Y (Fig. 4e–i). It should be noted that both F286^6.58^ and F290^6.62^ are located near the extracellular surface of the receptor, whereas F279^6.51^, F309^7.39^, and F312^7.42^ are located in the bottom of the ligand-binding pocket (Fig. 2d). These observations suggest that both F286^6.58^ and F290^6.62^ play a role in the entry of ghrelin into the receptor core, whereas F279^6.51^, F309^7.39^, and F312^7.42^ contribute to recognition of the acyl-modification of ghrelin. A recent study combining NMR with molecular modeling supports the concept that the n-octanoyl moiety of ghrelin is essential for access to the ligand-binding pocket^34^.

**Fig. 5:**
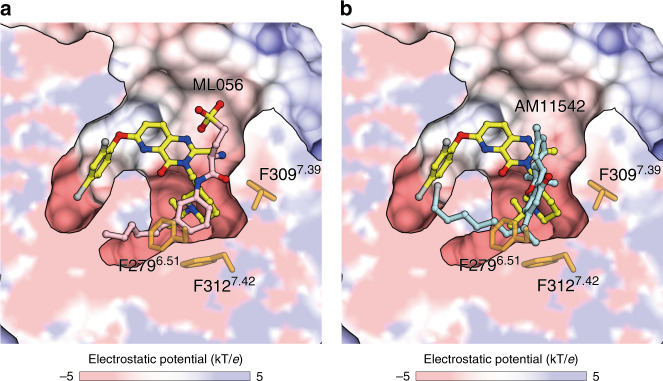
Comparison of ligand–binding modes of the ghrelin receptor, S1P_1_ receptor, and CB_1_ receptor. The ghrelin receptor surface and its cross-section were colored according to electrostatic potential from red (negative) to blue (positive) using APBS tools. **a** The S1P_1_ receptor (PDB ID: 3V2Y) and (**b**) the CB_1_ receptor (PDB ID: 5XRA) are superposed onto the ghrelin receptor. The 279^6.51^, F309^7.39^, and F312^7.42^ of the ghrelin receptor and Compound 21 are depicted as orange and yellow sticks, respectively. The sphingolipid mimic S1P_1_ antagonist (ML056) and the CB_1_ antagonist (AM11542) are depicted as pink and cyan sticks, respectively.

Meanwhile, we found that I178^4.60^, L181^4.63^, and M213^5.39^ in cavity II are not important for the interaction with ghrelin, as their alanine mutations caused no decrease in ghrelin-induced activity, although the basal receptor activities were suppressed (Supplementary Fig. 10a–c). In fact, we found that des-acyl ghrelin not only was unable to activate the ghrelin receptor, but also did not bind to the ghrelin receptor at all (Supplementary Fig. 11), suggesting that the acyl-modification is necessary for ghrelin to enter the binding pocket of the ghrelin receptor. An in silico docking study of inverse agonists to a homology model of ghrelin receptor also suggested that F279^6.51^, F309^7.39^, and F312^7.42^ are key residues for receptor activation/inactivation^35^. For other lipid receptors, the equivalent cavities contain multiple phenylalanine residues and accommodate hydrophobic moieties of their lipo-ligands. For example, the acyl tail of ML056, an antagonist of the S1P_1_ receptor, and both the tricyclic tetrahydrocannabinol ring and alkyl chain of AM11542, an antagonist of the CB_1_ receptor, are placed in the cavity (Fig. 5a, b)^26,36^. These facts suggest that the acyl moiety of ghrelin could be located in the cavity, where it interacts with phenylalanine residues essential for receptor activation.

## Discussion

This study provides insights into the interactions between the ghrelin receptor and ghrelin, and our findings may explain why the acyl-modification of the ghrelin peptide is necessary for ghrelin receptor activation. Several ghrelin mimetics are under development for treatment of cancer cachexia or metabolism-linked disorders, and our results will promote the design of more potent and effective ghrelin mimetics.

The ghrelin receptor may have properties of both peptide hormone GPCRs and lipid GPCRs. As described above, the binding pocket of the ghrelin receptor is bifurcated into two cavities, in which cavity I is more important for the binding of ghrelin, as determined by the results of mutagenesis analyses. In the peptide hormone GPCRs, such as NTSR1, ET_B_R, and AT_2_ receptor, the polar amino acids in the ligand binding pockets are important for recognition of the peptide cores of the endogenous ligands. In the case of the ghrelin receptor, the polar amino acids in cavity I is the core for recognition of the peptide part of ghrelin, because the alanine mutations of the polar amino acids in the cavity I abolish receptor activity (Fig. 4a–d).

On the other hand, in many lipid GPCRs, a wide gap between transmembrane bundles is proposed to serve as the access route for lipid ligands. However, in the case of the ghrelin receptor, the gap structure (crevasse) is probably not the access route for ghrelin into the ligand binding pocket, but instead seems to be the recognition site for the acyl-modification of ghrelin. This idea is supported by the results of hydrophobic amino acid mutations in the gap region, in particular mutations of the phenylalanine residues such as F279^6.51^, F309^7.39^, and F312^7.42^. In the S1P_1_ and CB_1_ receptor, which are known as typical lipid GPCRs, the position of the ligand-binding pockets is equivalent to the position of cavity I in the ghrelin receptor (Fig. 5a, b)^26,36^. Thus, in the ghrelin receptor, the hydrophobic environment of the ligand-binding pocket seems to accommodate the acyl acid moiety of ghrelin, just as lipid GPCRs accommodate hydrophobic moieties of their lipo-ligands.

Furthermore, the gap structure of the ghrelin receptor is composed of TM6 and TM7, whose movements are thought to change the GPCR conformation into its active form^37^. Thus, the interaction between the gap structure and the acyl acid moiety of ghrelin may participate in transforming the ghrelin receptor into an active conformation. Further details, obtained by structural analysis of the active form of ghrelin receptor bound to acyl-modified ghrelin, will be required to fully elucidate the mechanism of ghrelin receptor activation.

## Methods

### Expression and purification of the ghrelin receptor

Human *GHSR* cDNA, which is based on the nucleotide sequence of the human *GHSR* gene (encoded by GHSR; UniProt accession Q92847), was cloned into a modified pFastBac1 vector (Invitrogen) containing an expression cassette with a hemagglutinin signal sequence and a FLAG epitope tag followed by a human rhinovirus (HRV) 3C protease recognition site at the N terminus, and an HRV 3C protease recognition site followed by an enhanced green fluorescent protein (eGFP) and an 8xHis tag at the C terminus. Twenty-eight amino acids of the N terminus (residues 1–28) were replaced with the thermostabilized apocytochrome b_562_RIL (bRIL) from *Escherichia coli* (M7W, H102I, and R106L) protein^38,39^, and twenty amino acids were deleted from the C terminus (residues 347–366). Two mutations were introduced: T130K to improve thermostability and expression, and N188Q to avoid glycosylation. Recombinant baculovirus was prepared using the Bac-to-Bac baculovirus expression system (Invitrogen). *Spodoptera frugiperda* (Sf9) insect cells (Thermo Fisher Scientific) were infected with baculovirus at a cell density of 2–3.5 × 10^6^ cells ml^−1^ at multiplicity of infection of 1 in PSFM-J1 medium (Wako) supplemented with 2%(v/v) fetal calf serum, 50 units ml^−1^ penicillin, 50 µg ml^−1^ streptomycin, and 0.5 µg ml^−1^ amphotericin B. Infected cells were harvested by centrifugation 48 h post-infection, and the cell pellets were stored at −80 °C for future use.

The cell pellets were homogenized using a Dounce homogenizer in hypotonic buffer containing 10 mM HEPES-NaOH (pH7.5), 20 mM KCl, 10 mM MgCl_2_, and EDTA-free protease inhibitor cocktail (Nacalai Tesque). The cell membranes were isolated by ultracentrifugation at 100,000 × *g* for 30 min at 4 °C. Washing of the membranes was performed by two rounds of Dounce homogenization and centrifugation in high-osmolarity buffer containing 10 mM HEPES-NaOH (pH7.5), 1.0 M NaCl, 20 mM KCl, and 10 mM MgCl_2_. Purified membranes were resuspended in a buffer containing 50 mM HEPES-NaOH (pH 7.5), 500 mM NaCl, 2 mg ml^−1^ iodoacetamide (Wako), and 5 µM YIL781 (Tocris), and incubated overnight at 4 °C in the dark. The membranes were then solubilized in a solubilization buffer containing 50 mM HEPES-NaOH (pH7.5), 500 mM NaCl, 1%(w/v) n-dodecyl-ß-D-maltopyranoside (DDM; Anatrace), 0.2% cholesteryl hemisuccinate (CHS; sigma), and 0.2% (w/v) sodium cholate (Dojindo) for 2 h at 4 °C, followed by ultracentrifugation at 100,000×*g* for 30 min at 4 °C. Solubilized supernatant supplemented with 5 mM imidazole was mixed with 10 ml of Ni-NTA agarose (Qiagen) in batch-binding mode for 2 h at 4 °C. After binding, the Ni resin was washed with 10 column volumes of Ni wash buffer containing 30 mM HEPES-NaOH (pH7.5), 750 mM NaCl, 0.1% DDM, 0.02% CHS, 0.02% sodium cholate, and 5 µM YIL781. The protein was eluted with three column volumes of Ni wash buffer containing 500 mM imidazole. Elution from the Ni resin was supplemented with 2 mM calcium and loaded onto M1 anti-FLAG affinity resin (Sigma). Detergent was gradually exchanged on the M1 resin from DDM to 0.01%(v/w) lauryl maltose neopentyl glycol (LMNG; Anatrace), and YIL781 was replaced with 5 µM Compound 21, a ghrelin receptor antagonist. The receptor was eluted with 3 column volumes of FLAG elute buffer containing 20 mM HEPES-NaOH (pH7.5), 100 mM NaCl, 0.01% LMNG, 0.001% CHS, 200 µg ml^−1^ Flag peptide (Peptide Institute Inc.), 5 mM EDTA, and 5 µM Compound 21. The N-terminal FLAG tag and C-terminal eGFP-8xHis tag were cleaved by 3 C protease overnight at 4 °C. The purified receptor was concentrated with a 50-kDa molecular weight cut-off Amicon Ultra concentrator (Millipore) and loaded onto Superdex 200 Increase 10/300 GL (GE Healthcare) in buffer containing 20 mM HEPES-NaOH (pH7.5), 100 mM NaCl, 0.01% LMNG, and 0.001% CHS. The peak fractions were collected and flash-frozen with liquid nitrogen and stored at −80 °C until use.

### Protein thermostability assay

The thermal stability analysis was performed with the thiol-specific fluorochrome N-[4-(7-diethylamino-4-methyl-3-coumarinyl)phenyl]maleimide (CPM)^40^. CPM at 4 mg ml^−1^ in DMSO was diluted 40-fold into buffer containing 20 mM HEPES-NaOH (pH 7.5), 100 mM NaCl, 0.01% LMNG, and 0.001% CHS. Purified protein (20 µg) was mixed with diluted CPM and incubated on ice for 15 min in darkness. The reaction mixture (20 µl) was heated in a controlled manner with a ramp rate of 3 °C min^−1^ in a MyiQ2 thermal cycler (Bio-Rad). The excitation wavelength was set at 387 nm, and the emission wavelength at 463 nm. Assays were performed over a temperature range starting from 4 °C and ending at 80 °C. The peak in the derivative of the fluorescence signal was used to determine the melting temperature.

### Antibody generation

All animal experiments described in this study conformed to the guidelines outlined in the Guide for the Care and Use of Laboratory Animals of Japan and were approved by Kyoto University Animal Care Committee (approval No. Med Kyo 16043). For use as antigen, purified human ghrelin receptor [29–346] (T130K/N188Q) was reconstituted into liposomes containing chicken egg yolk phosphatidylcholine (eggPC, Avanti) and monophosphoryl lipid A (Sigma-Aldrich). Briefly, MRL/lpr mice were immunized three times at 10–14-day intervals with 100 µg purified ghrelin receptor mutant. The immunized mice were sacrificed, and single-cell suspensions were prepared from their spleens. These cells were fused with NS-1 myeloma cells using polyethylene glycol (PEG) according to conventional methods. To screen for antibodies that specifically recognize native receptors, we developed an ELISA method using proteoliposomes^41^. For liposome ELISA, we used purified ghrelin receptor mutant reconstituted into liposomes containing biotinyl phosphatidylethanolamine (Avanti) to maintain the protein in its native conformation and effectively immobilize liposomes onto Streptavidin-coated plates (Nunc). To eliminate antibodies recognizing the flexible loops, the N and C termini, or unstructured regions of the ghrelin receptor, we performed ELISA using ghrelin receptor denatured with 1% (w/v) SDS. The selected cells were isolated by limiting dilution to establish monoclonal hybridoma cell lines producing antibodies against the ghrelin receptor. The supernatant of the large-scale cell culture was loaded onto HiTrap Protein G HP (GE Health care) to purify mouse IgG. Fab fragments were obtained by proteolytic cleavage of IgG with papain (Worthington) and purified by Superdex200 10/60 (GE Healthcare). Purified Fab monomer (Fab 7881) was concentrated to 10 mg ml^−1^ using a 10-kDa molecular weight cut-off Amicon Ultra concentrator. Sequences of Fab fragments were determined according to the standard 5’-RACE method using total RNA isolated from hybridoma cells. The Fab 7881 obtained in this manner was used for crystallization of the ghrelin receptor–Fab complex.

### Crystallization of the ghrelin receptor–Fab complex

Purified ghrelin receptor and the Fab 7881 were mixed in a 1:1.5 molar ratio and incubated for 1 h at 4 °C. The complex was purified by gel filtration using Superdex 200 Increase 10/300 GL. Compound 21 was added to the peak fractions at a 1:5 molar ratio, and the mixture was concentrated to >30 mg ml^−1^ using a 50-kDa molecular weight cut-off Amicon Ultra concentrator. The purified ghrelin receptor–Fab 7881 complex was reconstituted into the lipidic cubic phase (LCP) by mixing with a monoolein:cholesterol mixture at a mass ratio of 10:1. In the mixture, the protein solution:lipid mass ratio was fixed at 2:3 using the twin-syringe mixing method. Crystallization experiments were carried out in 96-well glass sandwich plates (Molecular Dimensions) on an NT8-LCP crystallization robot (Formulatrix) using a 30 nl protein solution cubic phase overlaid with 600 nl precipitant solution, and incubated at 20 °C. The best crystals were obtained from precipitant solution containing 100 mM MES (pH 6.6–7.0), 400 mM potassium acetate, and 36–40% polyethylene glycol (PEG) 300. The crystals were collected within 2 weeks using mesh grid loops (WAKENBTECH) and flash-cooled in liquid nitrogen. Initial crystals were obtained in hanging drops by vapor diffusion at 20 °C from the precipitant solution containing 100 mM bicine (pH 9.0), 100 mM sodium chloride, and 30% PEG550MME.

### Data collection and structure determination

All diffraction datasets were collected at SPring-8 BL32XU^42^ using a micro-focused X-ray beam. Loop-harvested microcrystals were identified using raster scan and analysis by SHIKA^43^. Small wedge data, each consisting of 4–6°, were collected from single crystals, and the collected datasets were processed automatically using KAMO^44^ using XDS^45^, followed by hierarchical clustering analysis using the correlation coefficients of the normalized structure amplitudes between datasets. Finally, a group of outlier-rejected datasets were scaled and merged using XSCALE^46^. The structure was determined by molecular replacement with the program PHASER^47^, using the receptor portions of the antagonist-bound orexin receptor (PDB ID: 4S0V), bRIL (PDB ID: 1M6T), and the Fab 7881 antibody (this study, ID: 6KS2) as the search models. The SMILES string of the ligand was generated by Ligand Builder in COOT^48^, and the geometry restraints were generated by eLBOW in PHENIX^49^. The electron density map and the structural model were iteratively refined and rebuilt using PHENIX and COOT^50^. Data collection and refinement statistics are summarized in Supplementary Table 1. All molecular graphics were prepared using Cuemol2 (http://www.cuemol.org/).

### Radioligand binding studies of the ghrelin receptor

Ligand binding assays were performed using membrane preparations from HEK293 cells (ATCC) transiently expressing wild-type or mutant ghrelin receptors. (The crystallization construct was expressed in Sf9 cells.) The ghrelin receptor–expressing cells were homogenized on ice in homogenization buffer containing 50 mM Tris-HCl (pH7.5), 5 mM EDTA, 5 mM MgCl_2_, and EDTA-free protease inhibitor cocktail. The homogenate was ultracentrifuged at 100,000×*g* for 30 min at 4 °C, and the pellet was washed twice with cold homogenization buffer. The protein concentration was determined using BCA Protein Assay (Thermo Fisher). All binding assays were conducted in ghrelin binding buffer containing 50 mM Tris-HCl (pH7.5), 5 mM EDTA, 5 mM MgCl_2_, and 1% (w/v) bovine serum albumin. For the saturation binding assay, 20 μg (5 µg for crystallization construct) of membrane homogenates were incubated on ice for 1 h with 0.039–5 nM [^125^I]-ghrelin (PerkinElmer) or 3.125–100 nM (3.125–50 nM for crystallization construct) [^3^H]-Compound 21 (Sekisui Medical) in the absence or presence of 5 μM unlabeled ghrelin or 100 μM unlabeled Compound 21. Bound [^125^I]-ghrelin was quantified on an AccuFLEX-γ 8010 (Hitachi). Bound [^3^H]-Compound 21 was quantified with an AccuFLEX LCS-8000 liquid scintillation counter (Hitachi) after addition of 2.5 ml of Clear-Sol liquid scintillation cocktail (Nacalai Tesque). Data were analyzed by nonlinear curve-fitting using the program GraphPad Prism 5. Binding data are reported as means ± s.d.

### Mutagenesis analyses of residues in the ligand recognition

Receptor activities of mutated ghrelin receptors were examined. Mutagenesis of the ghrelin receptor was introduced using the QuikChange Site-Directed Mutagenesis Kit (Agilent). Full-length human ghrelin receptor was used for the mutagenesis. The positions of mutated amino acids and primers are listed in Supplementary Table 2. Each mutant ghrelin receptor was expressed in CHO cells, and its receptor-specific responses to ghrelin were examined using an intracellular Ca^2+^ concentration assay.

### Intracellular Ca^2+^ concentration assay

For the fluorescence-based assay to detect changes in intracellular Ca^2+^ concentration, CHO cells (ATCC) were seeded in 6-cm dishes at a density of 3 × 10^5^ cells/dish and transfected with the ghrelin receptor N188Q (control) or the indicated ghrelin receptor mutant. Transfections were performed using the FuGENE HD transfection reagent (Promega). The next day, the transfected cells were collected and plated into 96-well black-wall assay microplates (Corning) at a density of 3 × 10^4^ cells/well. After a 1-day incubation, the cells were incubated for 1 h with 100 μl of calcium indicator Fluo-4 from the FLIPR Calcium 5 Assay Kit (Molecular Devices) in Hank’s BSS containing 20 mM HEPES-NaOH (pH7.5) and 2.5 mM probenecid. Fifty-microliter aliquots of samples were tested to induce changes in fluorescence due to increased calcium concentration on a FlexStation3 (Molecular Devices). Maximum [Ca^2+^]_i_ changes were determined in quadruplicate (*n* = 4).

### Cell-surface expression levels of ghrelin receptors

HEK293 cells (ATCC) were seeded in 6-well plates at a cell density of 8 × 10^5^ cells/well and cultured for 24 h. The cells were transfected with complex of vector DNA (the FLAG-tagged N188Q ghrelin receptor (control) or the indicated FLAG-tagged ghrelin receptor mutants) and FuGENE HD transfection reagent (Promega). The next day, transfected HEK293 cells were harvested in PBS containing 1 mM EDTA, and then incubated for 30 min on ice with 2.5 μg ml^−1^ anti-FLAG antibody (Wako) and 5 μg ml^−1^ Alexa Fluor 488–conjugated anti-mouse IgG goat polyclonal antibody (Thermo Fisher Scientific) in FACS buffer (PBS, 2% FBS, 0.05% NaN_3_). Cell-surface expression levels were evaluated by flow cytometry on a Guava EasyCyte Plus Flow Cytometer. FLAG-positive cells were defined as cell populations with signals greater than the top 5% of MOCK cells.

### Luciferase assay

To detect constitutive activity of ghrelin receptor, CHO cells were seeded in 6-cm dishes at a density of 2.5 × 10^5^ cells/dish and cotransfected with pGL4.30[luc2P/NFAT-RE/Hygro] (2.5 μg, Promega), pRL-TK (0.1 μg, Promega), and the indicated ghrelin receptor plasmid (5 μg). Transfections were performed using the FuGENE HD transfection reagent (Promega). The next day, the transfected cells were collected and plated into 96-well white microplates (Corning) at a density of 2.5 × 10^4^ cells/well. After a 1-day incubation, the luciferase activities were determined using Dual-Luciferase Reporter Assay System (Promega). When assessing Compound 21 activity, the transfected cells were incubated with various concentrations of Compound 21 for 1 h just before luciferase activity measurement. All firefly luciferase activities were normalized against *Renilla* luciferase activity. All experiments were performed in sextuplicate.

### Statistical analysis

Data are presented as means ± s.e.m. The outliers on the luciferase activity were detected and removed based on the Smirnov–Grubbs test. The GraphPad Prism 8 software (GraphPad Software Inc.) was used for statistical analysis. Differences between groups were analyzed using the two-tailed Student *t* test. *P* < 0.05 was considered statistically significant.

### Reporting summary

Further information on research design is available in the Nature Research Reporting Summary linked to this article.

## Supplementary information

Supplementary Info

Peer Review File

Reporting Summary

## Data Availability

Data supporting the findings of this manuscript are available from the corresponding authors upon reasonable request. A reporting summary for this Article is available as a Supplementary Information file. The structure and corresponding structure factors have been deposited into the Protein Data Bank with the PDB 6KO5 for the human ghrelin receptor–Fab 7881 complex and PDB 6KS2 for Fab 7881. Source data are provided with this paper.

## Source data

Source Data File
