# Supplementary material for: Structure of an antagonist-bound ghrelin receptor reveals possible ghrelin recognition mode
Source: Nat Commun. 2020 Aug 19;11:4160. doi: 10.1038/s41467-020-17554-1 (PMC7438500; doi:10.1038/s41467-020-17554-1)
Supplement: Supplementary file 1 — Supplementary Info [file 41467_2020_17554_MOESM1_ESM.pdf]

Supplementary Information

Structure of an antagonist-bound ghrelin receptor reveals possible ghrelin recognition mode

Shiimura et al.

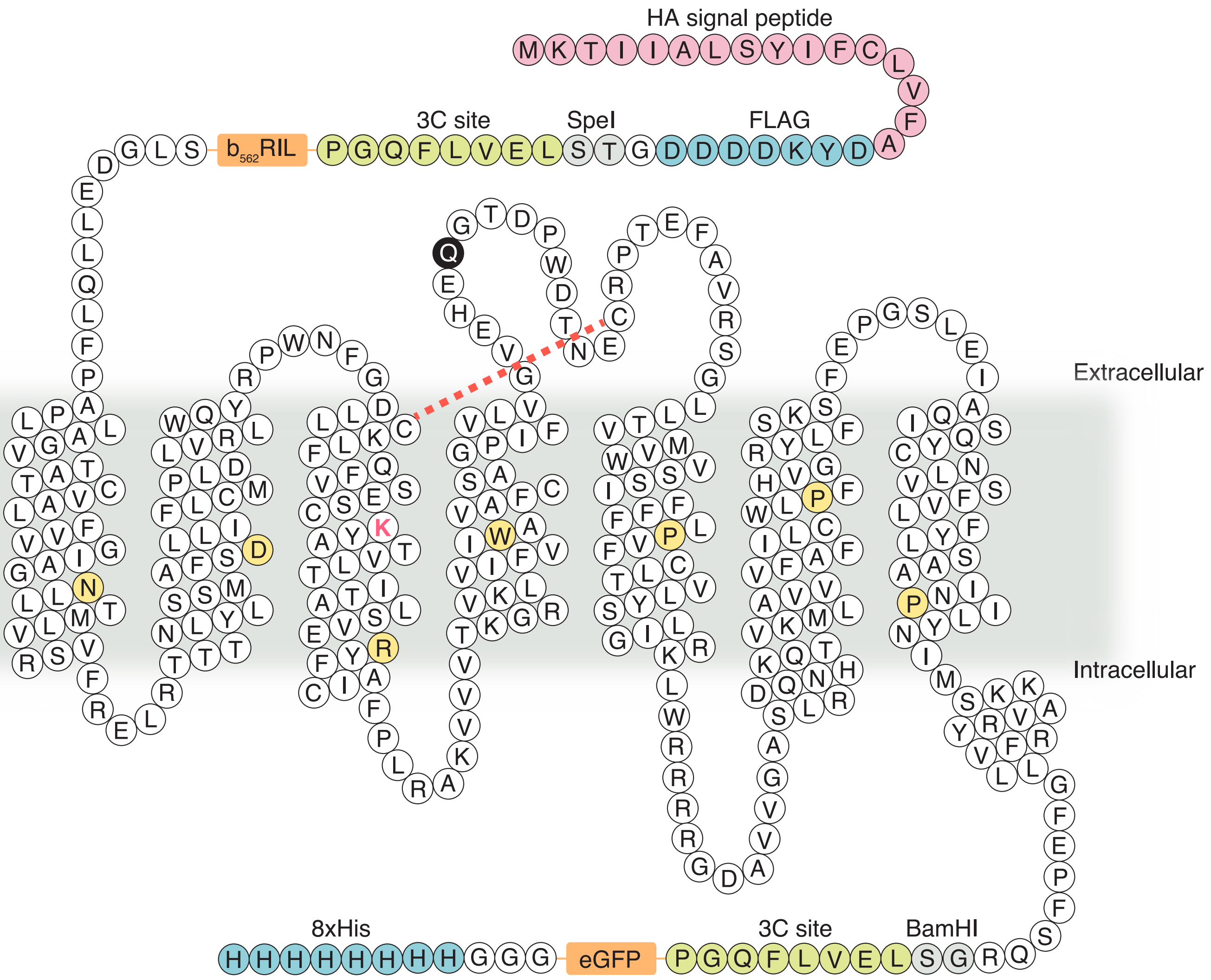

Supplementary Figure 1

Crystallization construct of the ghrelin receptor.

Snake-plot diagram of the crystallization construct of the ghrelin receptor. The bRIL was introduced at the N terminus to stabilize the receptor. The HA signal peptide (magenta) at the N terminus was introduced to enhance expression. To facilitate protein purification, a FLAG affinity tag was introduced at the N terminus (cyan), and a 8x His tag was introduced at the C terminus (cyan) along with a human rhinovirus (3C) cleavage site (green). A potential N-glycosylation site (N188) was replaced with glutamine (white letter in the black circle). The N-terminal region (residues 1–28) and the C-terminal region (residues 347–366) were deleted to facilitate crystallization. A mutation (T130K) shown in red letters was introduced to facilitate expression in Sf9 cells. The dashed red line indicates a disulfide bond between Cys116$^{3.28}$ and Cys198^{ECL2}. The X.50 position (Ballesteros–Weinstein numbering) of each transmembrane helix is shown by yellow circles. eGFP was introduced in the C-terminal region to monitor expression and purification.

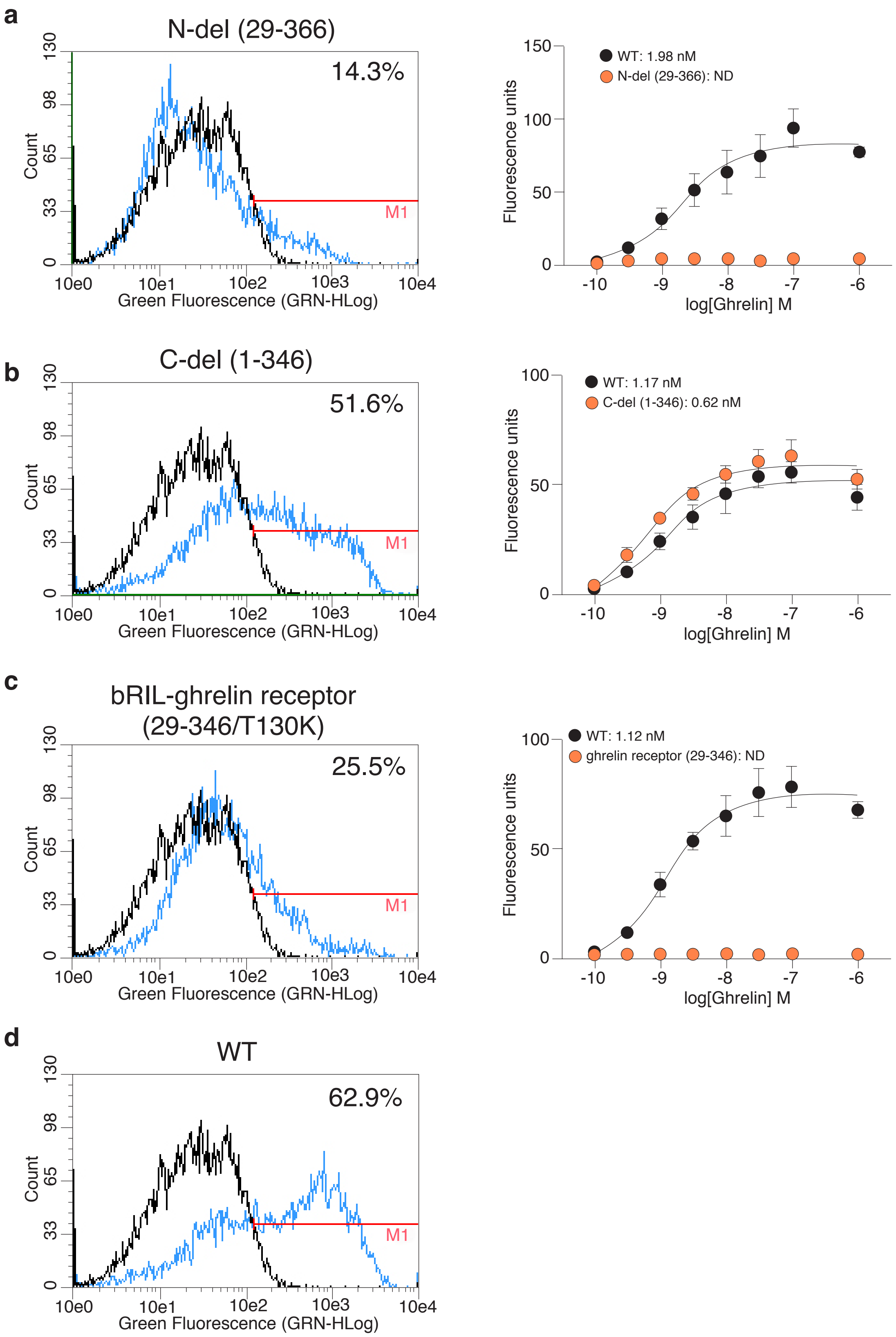

Supplementary Figure 2

Surface expressions and receptor activities of WT and ghrelin receptor constructs.

a, Deletion of the N-terminal 28-amino acids. **b**, Deletion of the C-terminal 20-amino acids. **c**, Cell surface expression of the construct used for crystallization (bRIL human ghrelin receptor (29-346/T130K)) and intracellular Ca^{2+} assay of ghrelin receptor (29-346). **d**, WT ghrelin receptor. Data from mock transfection and transfection with ghrelin receptors are shown as black and blue histograms, respectively. Marker M1 designats FLAG positive events. The M1-gated percentage is indicated in the upper-right. Receptor activities were measured by intracellular Ca^{2+} assay. EC50 values are means ± s.e.m. (n=4).

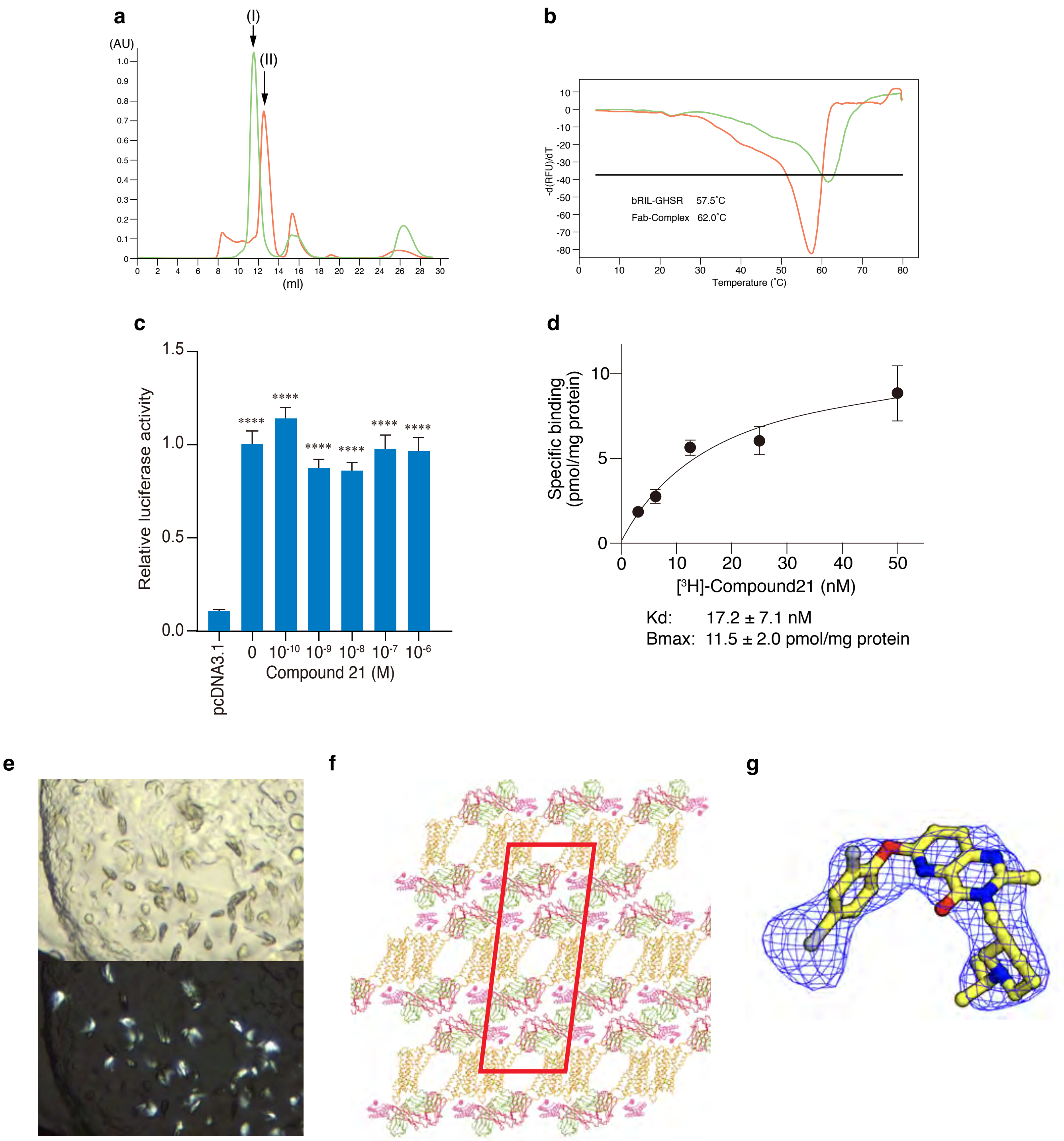

Supplementary Figure 3

Characterization of the ghrelin receptor–Compound 21–Fab 7881 complex.

a, Analytical size exclusion chromatography profile. (I) bRIL–human ghrelin receptor (hGHSR)–Fab 7881 complex (green) and (II) bRIL–hGHSR (red). **b**, Thermal stability analysis of bRIL–hGHSR–Fab 7881 complex (green) and bRIL–hGHSR (red). Compound 21 is present in the both samples. Fab 7881 increased Tm by about 5°C. **c**, Basal ghrelin receptor activity in the presence of Compound 21, as determined by luciferase assay. Data are represented as means ± s.e.m. (n=5–6). ****P<0.0001 vs. pcDNA3.1. **d**, Specific binding of [^{3}H]-Compound 21 to the ghrelin receptor construct. **e**, Crystal images of the bRIL–hGHSR–Fab 7881 complex. **f**, Crystal packing of the bRIL–hGHSR–Compound 21–Fab 7881 complex. The ghrelin receptor is shown in cartoon representation and colored in orange. The bRIL fusion protein is shown in pink cartoon representation. For Fab 7881, the crimson and green-yellow cartoons represent the heavy and light chains, respectively. Compound 21 is colored in yellow. **g**, The omitted mFo–DFc electron density (blue mesh) of Compound 21 contoured at 3.0 σ (Polder map), implemented in the PHENIX software suite.

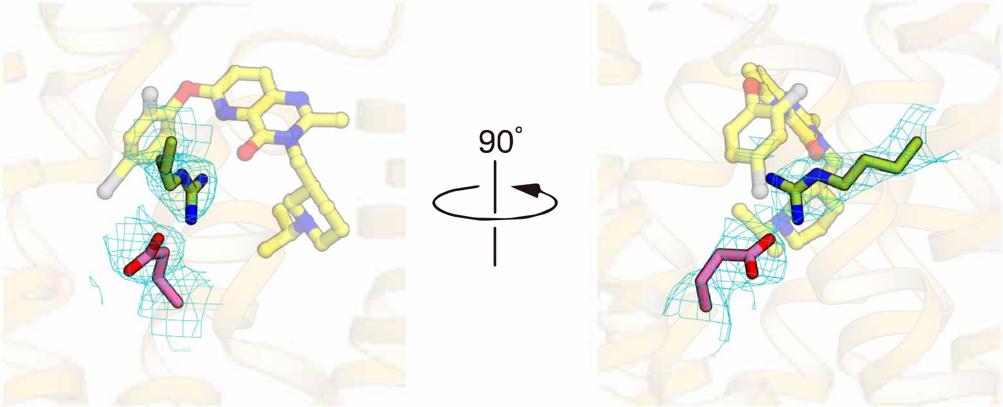

Supplementary Figure 4
Electron density of the E124–R283 interaction.
The electron density 2mFo–DFc map (cyan mesh) of the interaction between E124$^{3.33}$ and R283$^{6.55}$ was calculated from the 3.3 Å resolution dataset contoured at 1.0 σ. The pink and green sticks represent E124$^{3.33}$ and R283$^{6.55}$, respectively.

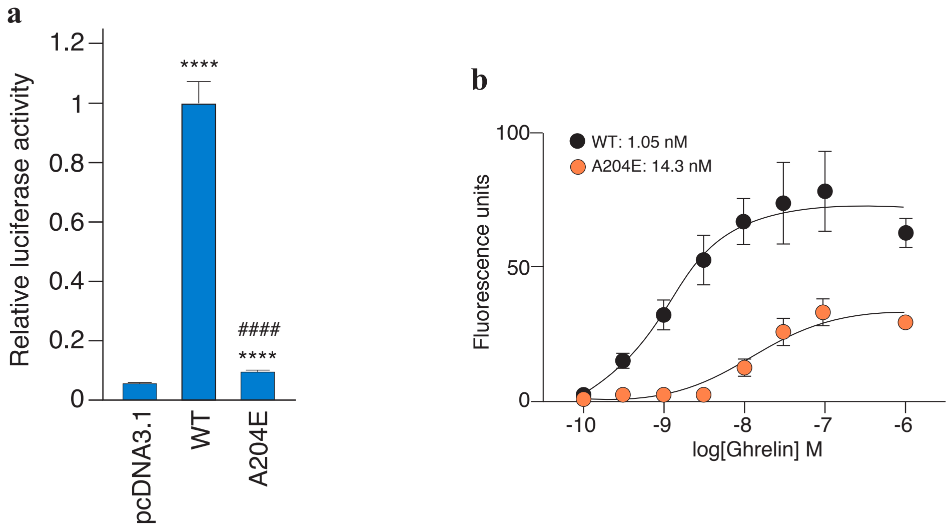

Supplementary Figure 5

Receptor activities of the A204E mutant

a, Basal receptor activity, as determined by luciferase assay. Basal receptor activity of the A204E mutant is significantly suppressed. ****P<0.0001 compared with the pcDNA3.1 ####P<0.0001 vs. WT. **b**, Receptor activities were examined by intracellular Ca^{2+} assay. EC50 values are means ± s.e.m. (n=4).

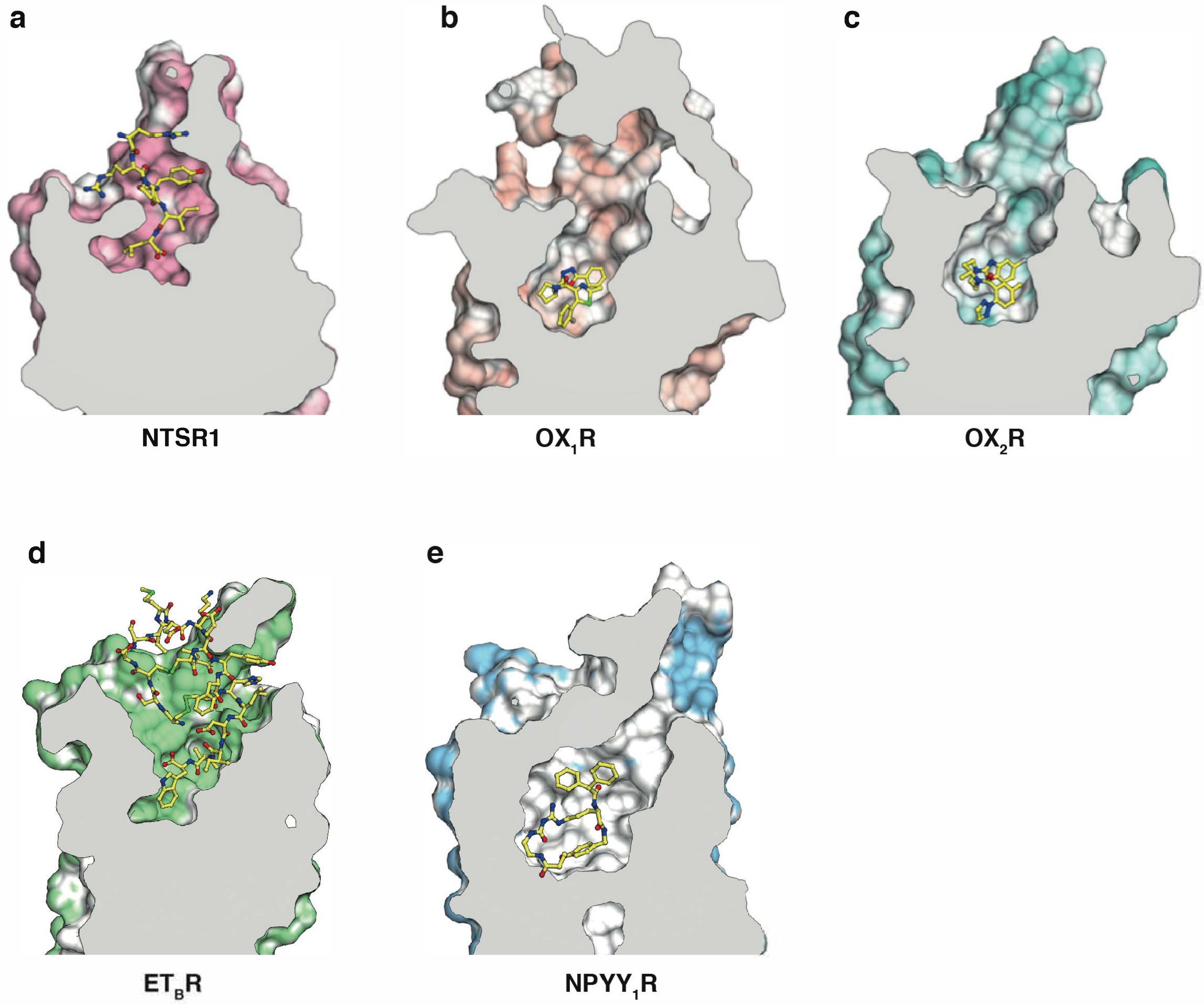

Supplementary Figure 6

Structural comparison of the orthosteric ligand-binding pocket of the peptide-ligand GPCRs.

(**a**) Neurotensin receptor NTSR1 (PDB ID: 4GRV), (**b**) orexin receptor OX_1R (PDB ID: 4ZJ8), (**c**) orexin receptor OX_2R (PDB ID: 4S0V), (**d**) endothelin type B receptor ETBR (PDB ID: 5GLH), and (**e**) neuropeptide Y receptor NPY Y_1R (PDB ID: 5ZBQ). Ligands are represented by sticks. Bifurcated ligand pocket is observed only in the ghrelin receptor.

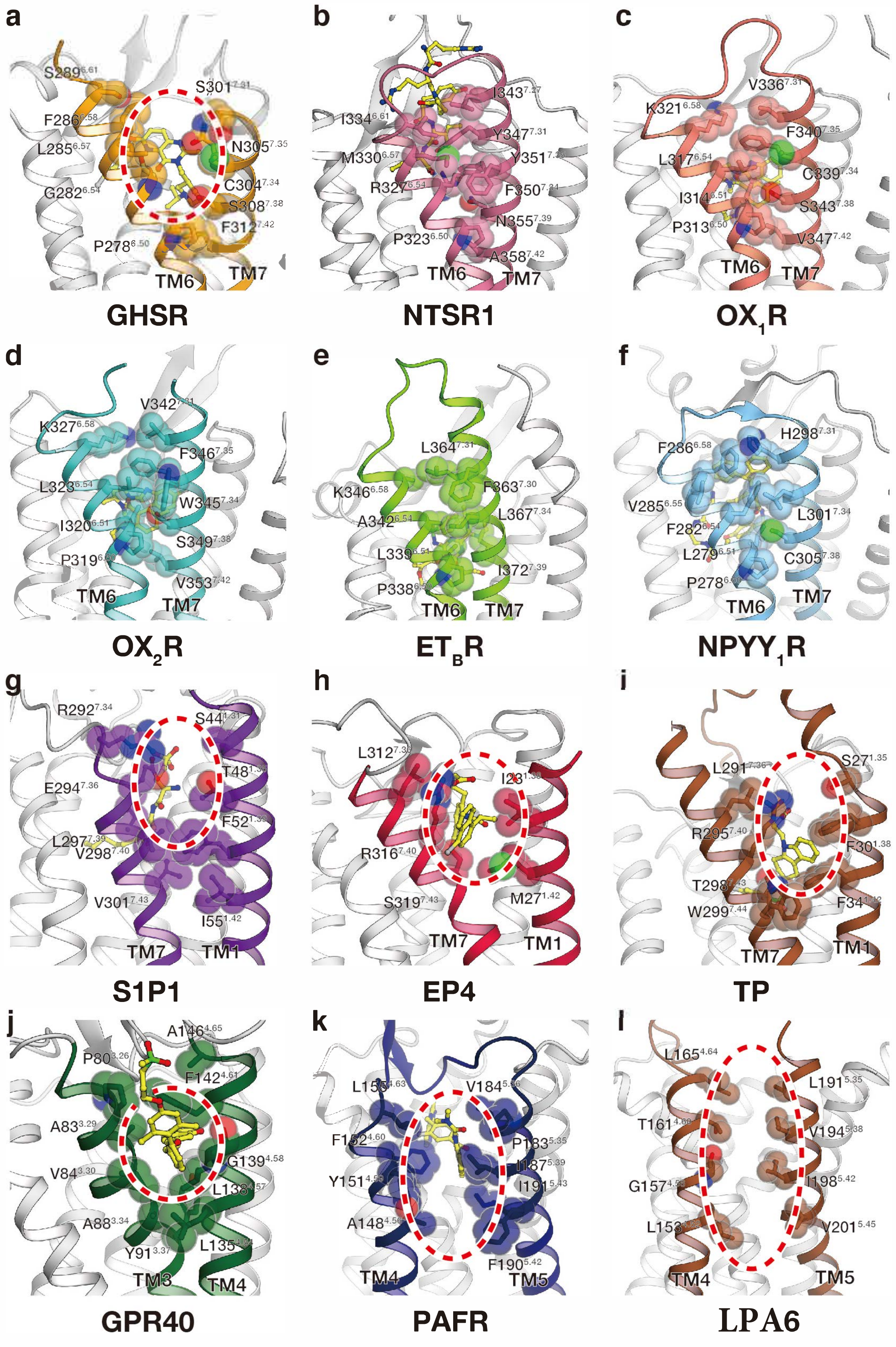

Supplementary Figure 7

Structural comparison of the crevasse regions in peptide hormone GPCRs and the gaps in lipid ligand GPCRs.

a, Ghrelin receptor (GHSR). The gap (crevasse) between TM6 and TM7 is indicated by the dotted red line. **b**, Neurotensin receptor NTSR1 (PDB ID: 4GRV). **c**, Orexin receptor OX$_1$R (PDB ID: 4ZJ8). **d**, Orexin receptor OX$_2$R (PDB ID: 4S0V). **e**, Endothelin receptor ETBR (PDB ID: 5GLH). **f**, Neuropeptide Y receptor NPY Y$_1$R (PDB ID: 5ZBQ). **g**, S1P$_1$ receptor (PDB ID: 3V2Y). **h**, EP4 receptor (PDB ID: 5YHL). **i**, Thromboxane (TP) A$_2$ receptor (PDB ID: 6IIU). **j**, GPR40 (PDB ID: 4PHU). **k**, PAF receptor (PDB ID: 5ZKQ). **l**, Lysophosphatidic acid receptor LPA6 (PDB ID: 5XSZ). Dotted red lines in **g-l** indicate the gaps for the lipid ligand access.

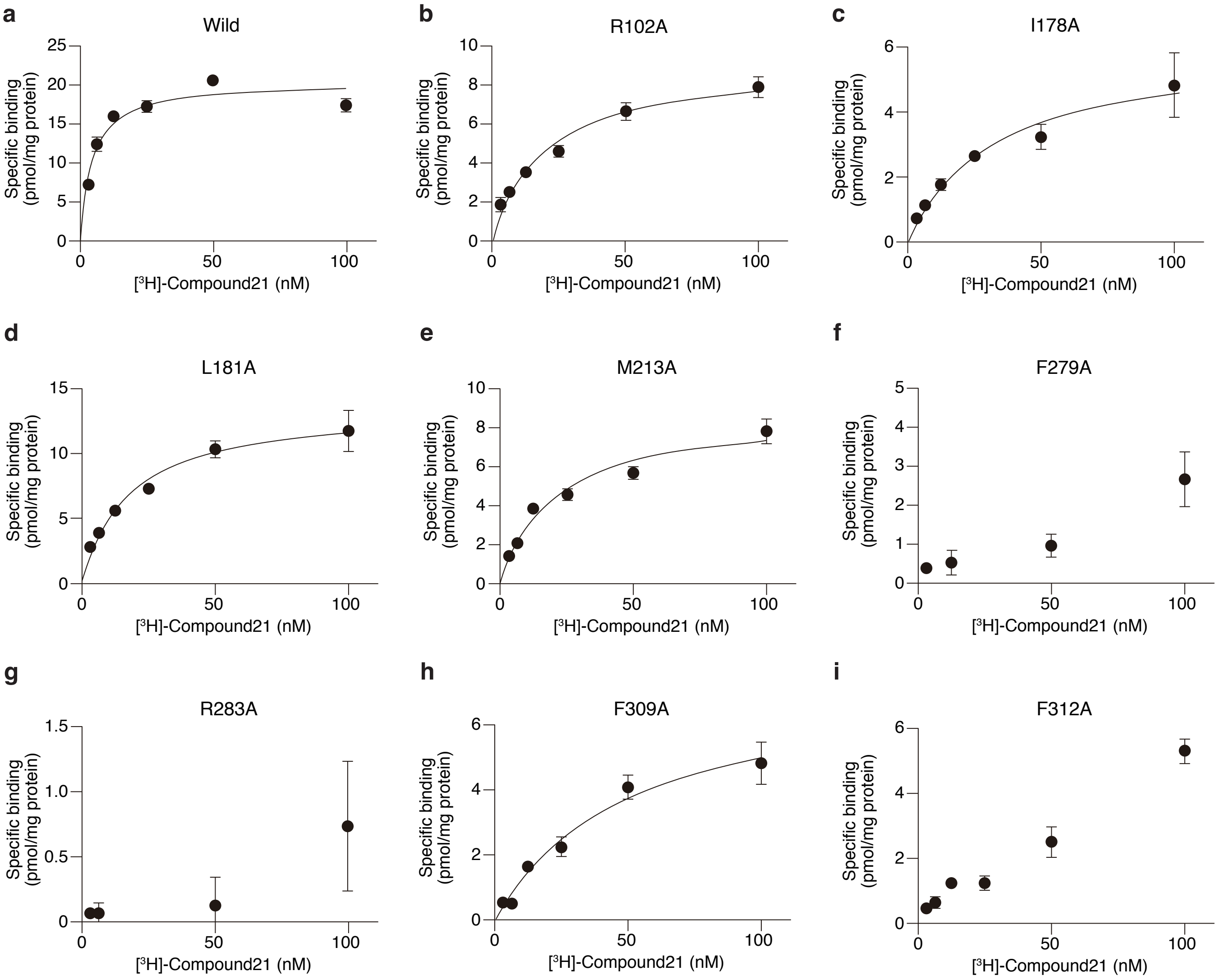

j

	Kd (nM)	*Bmax* (pmol/mg protein)
Wild	4.42 ± 0.76	20.4 ± 3.8
$R102^{2.63}A$	18.9 ± 3.3	9.2 ± 0.6
$I178^{4.60}A$	31.9 ± 11.7	6.0 ± 0.9
$L181^{4.63}A$	17.1 ± 3.5	13.6 ± 1.0
$M213^{5.39}A$	20.4 ± 0.8	8.8 ± 0.6
$F279^{6.51}A$	ND	ND
$R283^{6.55}A$	ND	ND
$F309^{7.39}A$	49.2 ± 14.9	7.4 ± 1.1
$F312^{7.42}A$	ND	ND

Supplementary Figure 8

Specific assays of [^{3}H]-Compound 21 binding to the wild-type and the mutant ghrelin receptors.

a–i, Amino acids located in the ligand binding cavities were mutated to an alanine residue. Each points represents mean ± s.d. from three independent experiments. Vertical axes show specific binding values of [^{3}H]-Compound 21. **j**, The corresponding *Kd* and *Bmax* values, which were calculated by fitting a nonlinear curve.

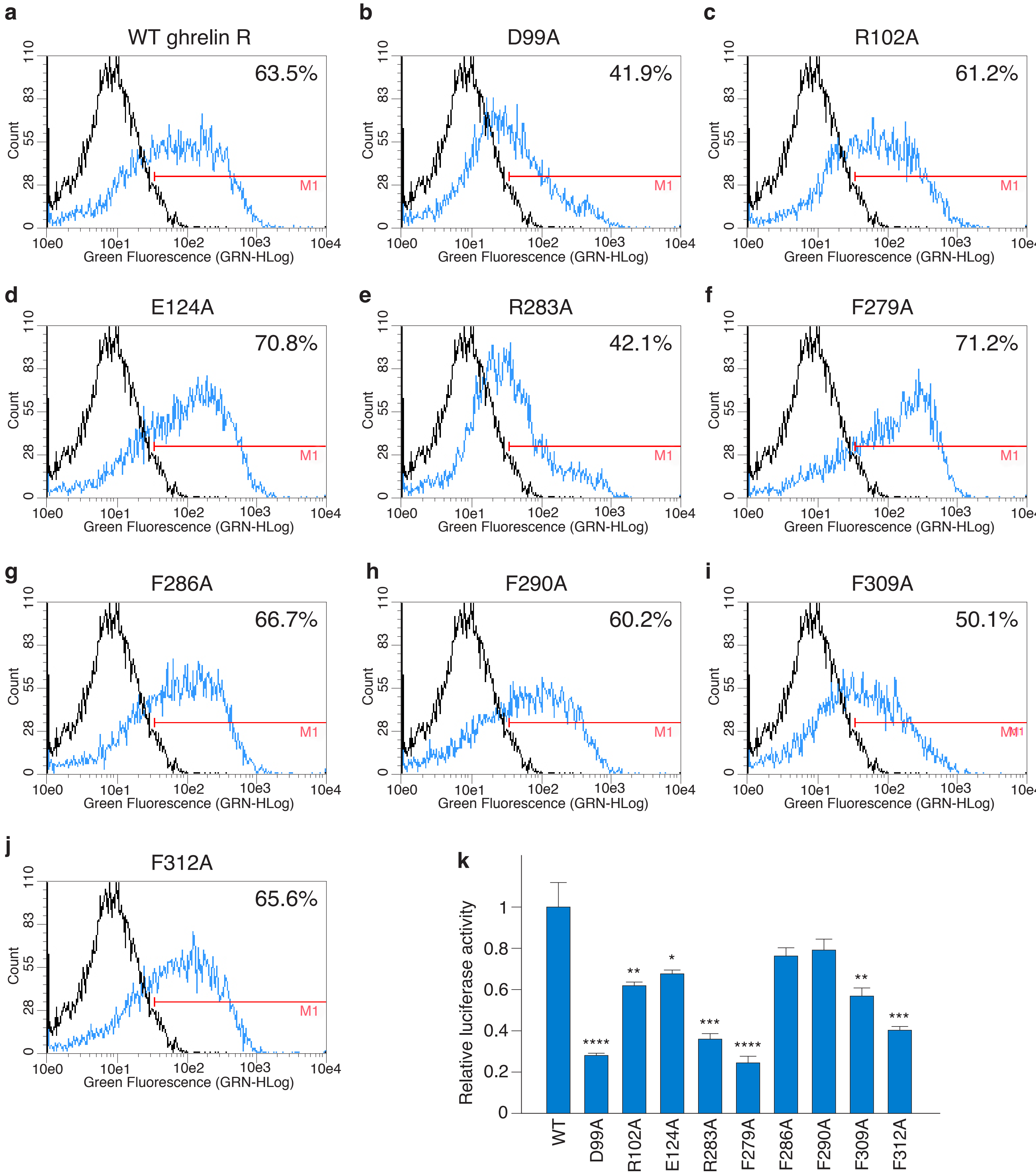

Supplementary Figure 9

Surface expression and basal activity of wild-type and mutant ghrelin receptors.

a–j, Surface expression of ghrelin receptors analyzed by flow cytometry. Mock transfection and ghrelin receptors are shown as black and blue histograms, respectively. Marker M1 designats FLAG positive events. The M1-gated percentage is described in the upper-right. The expression levels were decreased by the D99A, R283A and F309A mutations. **k**, Basal receptor activity measured by luciferase assay. Data are represented as mean ± s.e.m. (n=5-6). *P<0.05; **P<0.01; ***P<0.001; ****P<0.0001, WT ghrelin receptor vs. mutants.

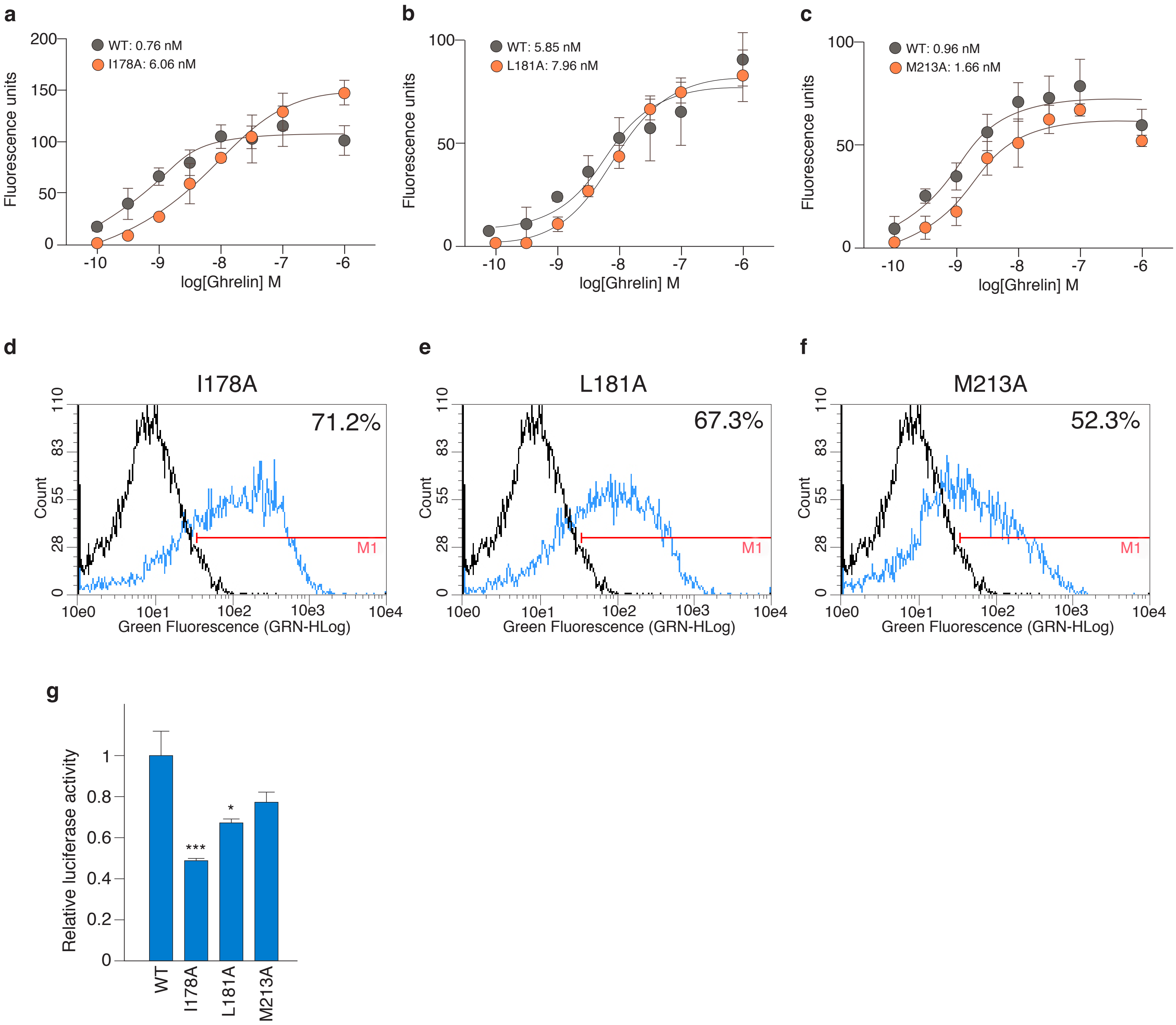

Supplementary Figure 10

Mutagenesis analyses of residues in the ligand-binding pocket, surface expression and basal activity of mutant ghrelin receptors.

a–c, Receptor activities were monitored by intracellular Ca^{2+} concentration assay. EC50 values are expressed as means ± s.e.m. (n=4). **d–f**, Surface expression of mutant ghrelin receptors. Data from mock transfection and transfection with ghrelin receptors are shown as black and blue histograms, respectively. Marker M1 designats FLAG positive events. The M1-gated percentage is described in the upper-right. Expression levels of M213A mutant decreased relative to the WT ghrelin receptor shown in Supplementary Figure 8. **g**, Basal receptor activity measured by luciferase assay. Data are represented as means ± s.e.m. (n=6). *P<0.05; ***P<0.001, WT ghrelin receptor vs. mutants. The activity of the WT ghrelin receptor is the same as in Extended Data Figure 8, because the luciferase assay was performed under the same conditions on the same day.

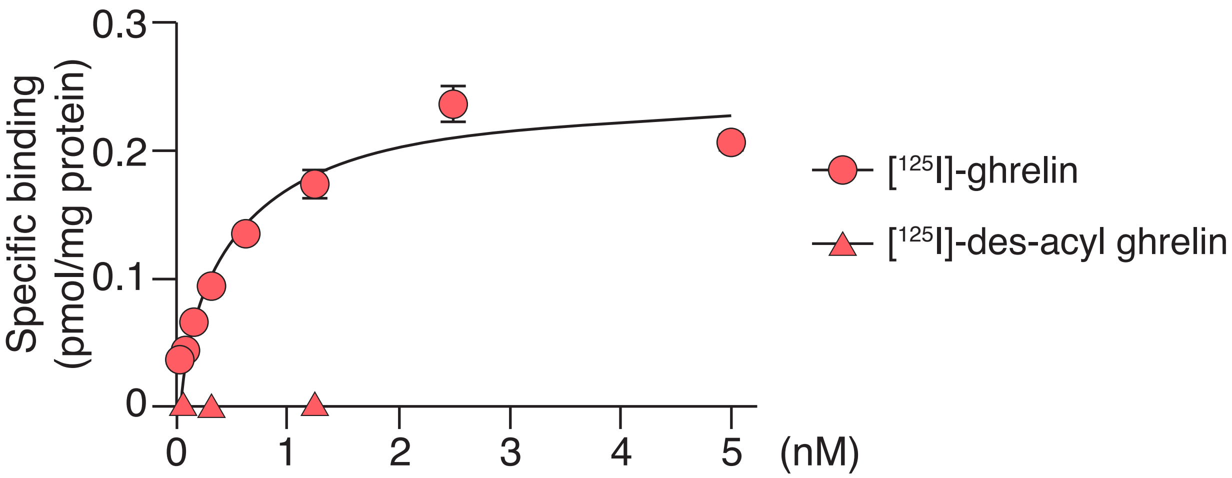

Supplementary Figure 11

Radioligand binding assays of the ghrelin receptor.

Binding assay of ghrelin (n=3) and des-acyl ghrelin (n=3). *Bmax* (pmol/mg protein) and *Kd* (nM) of ghrelin are 0.25 and 0.45, respectively. Three values of des-acyl ghrelin were too low to calculate the specific bindings.

Supplementary Table 1

Protein name	GHSR–Fab complex (PDB ID: 6KO5)	Fab_7881 (PDB ID: 6KS2)
X-ray data collection		
Source, wavelength	SPring-8 BL32XU, 1.0 Å	SPring-8 BL32XU, 1.0 Å
Resolution (Å)[a]	49.4–3.3 (3.4–3.3)	47.5–1.75 (1.86–1.75)
Space group	*C*2	$P2_12_12_1$
Unit cell parameter		
a, b, c (Å)	253.76, 44,75, 94.40	42.24, 107.92, 189.89
α, β, γ (°)	90.00, 97.26, 90.00	90.00, 90.00, 90.00
Unique reflections[a]	16364 (1638)	87539 (13650)
Redundancy[a]	36.2 (36.6)	6.8 (7.0)
Completeness[a]	99.9 (100.0)	99.2 (97.2)
R_{merge} (%)[a]	87.4 (511.2)	11.2 (109.3)
$CC_{1/2}$ (%)[a]	99.2 (62.6)	99.7 (75.2)
$\langle I/\sigma(I)\rangle$	10.3 (1.5)	9.8 (1.3)
Refinement		
Resolution (Å)	49.4–3.3	47.5–1.75
R_{work}/R_{free} (%)[b]	21.1 / 25.8	19.2 / 23.3
R.m.s. deviations		
Bond (Å)	0.002	0.011
Angles (°)	0.505	1.215
No. of atoms [average *B*-factors (Å^2)]		
bRIL-GHSR	3168 (90.8)	-
Compound21	31 (66.6)	-
Fab fragment	3344 (72.7)	6665 (39.8)
Water	-	620 (42.3)
Ramachandran plot		
Favored region (%)	97.0	97.6
Allowed region (%)	2.9	2.1
Outlier (%)	0.1	0.2

[a]Values for the highest resolution shells are shown in parentheses.

[b]R_{work} was calculated with 95% of the unique reflections used for refinement, whereas R_{free} was calculated with the remaining 5% of the unique reflections.

Supplementary Table 2: Primers for site-directed mutagenesis

Oligonucleotides primer	Forward	Reverse
GHSR_D99A	CCCCTGGCCCTCGTTCGCCTCTGGCAG	AACGAGGGCCAGGGGCATGCAGAGGAA
GHSR_D99E	CCCCTGGAGCTCGTTCGCCTCTGGCAG	AACGAGCTCCAGGGGCATGCAGAGGAA
GHSR_R102A	CTCGTTGCCCTCTGGCAGTACCGCCCC	CCAGAGGGCAACGAGGTCCAGGGGCAT
GHSR_R102K	CTCGTTAAGCTCTGGCAGTACCGCCCC	CCAGAGCTTAACGAGGTCCAGGGGCAT
GHSR_E124A	GTCAGTGCCAGCTGCACCTACGCCACG	GCAGCTGGCACTGACGAATTGGAAGAG
GHSR_E124D	GTCAGTGACAGCTGCACCTACGCCACG	GCAGCTGTCACTGACGAATTGGAAGAG
GHSR_I178A	TTCTGCAGCGCCGGACCCGCTTTCGTG	CACTCCGACGAGCACGAAAGCGGGTCC
GHSR_L181A	GCCGGACCCATCTTCGTGGCTGTCGGA	CTCGTGCTCCACTCCGACAGCCACGAA
GHSR_M213A	TCTGGACTGCTCACGGTCGCTGTGTGG	GATGCTGGACACCCACACAGCGACCGT
GHSR_F279A	CTCCCCGCCCACGTAGGACGCTATTTG	TACGTGGGCGGGGAGCCAGCAGAGGAT
GHSR_F279Y	CTCCCCTACCACGTAGGACGCTATTTG	TACGTGGTAGGGGAGCCAGCAGAGGAT
GHSR_R283A	GTAGGAGCCTATTTGTTTTCCAAATCC	CAAATAGGCTCCTACGTGGAAGGGGAG
GHSR_R283K	GTAGGAAAGTATTTGTTTTCCAAATCC	CAAATACTTTCCTACGTGGAAGGGGAG
GHSR_F286A	TATTTGGCCTCCAAATCCTTTGAGCCT	TTTGGAGGCCAAATAGCGTCCTACGTG
GHSR_F286Y	TATTTGTACTCCAAATCCTTTGAGCCT	TTTGGAGTACAAATAGCGTCCTACGTG
GHSR_F290A	AAATCCGCCGAGCCTGGCTCCTTGGAG	AGGCTCGGCGGATTTGGAAAACAAATA
GHSR_F290Y	AAATCCTACGAGCCTGGCTCCTTGGAG	AGGCTCGTAGGATTTGGAAAACAAATA
GHSR_F309A	GTGTCCGCCGTCCTCTTCTACCTCAGT	GAGGACGGCGGACACGAGGTTGCAGTA
GHSR_F309Y	GTGTCCTACGTCCTCTTCTACCTCAGT	GAGGACGTAGGACACGAGGTTGCAGTA
GHSR_F312A	GTCCTCGCCTACCTCAGTGCTGCCATC	GAGGTAGGCGAGGACAAAGGACACGAG
GHSR_F312Y	GTCCTCTACTACCTCAGTGCTGCCATC	GAGGTAGTAGAGGACAAAGGACACGAG
