# Supplementary material for: Structure of an antagonist-bound ghrelin receptor reveals possible ghrelin recognition mode
Source: Nat Commun. 2020 Aug 19;11:4160. doi: 10.1038/s41467-020-17554-1 (PMC7438500; doi:10.1038/s41467-020-17554-1)
Supplement: Supplementary file 3 — Reporting Summary [file 41467_2020_17554_MOESM3_ESM.pdf]

nature research

Corresponding author(s): Masayasu Kojima

Last updated by author(s): 2020-7-8

# Reporting Summary

Nature Research wishes to improve the reproducibility of the work that we publish. This form provides structure for consistency and transparency in reporting. For further information on Nature Research policies, see Authors & Referees and the Editorial Policy Checklist.

## Statistics

For all statistical analyses, confirm that the following items are present in the figure legend, table legend, main text, or Methods section.

| n/a | Confirmed | |
|---|---|---|
| ☐ | ☒ | The exact sample size (*n*) for each experimental group/condition, given as a discrete number and unit of measurement |
| ☒ | ☐ | A statement on whether measurements were taken from distinct samples or whether the same sample was measured repeatedly |
| ☐ | ☒ | The statistical test(s) used AND whether they are one- or two-sided<br>*Only common tests should be described solely by name; describe more complex techniques in the Methods section.* |
| ☐ | ☒ | A description of all covariates tested |
| ☒ | ☐ | A description of any assumptions or corrections, such as tests of normality and adjustment for multiple comparisons |
| ☐ | ☒ | A full description of the statistical parameters including central tendency (e.g. means) or other basic estimates (e.g. regression coefficient) AND variation (e.g. standard deviation) or associated estimates of uncertainty (e.g. confidence intervals) |
| ☐ | ☒ | For null hypothesis testing, the test statistic (e.g. *F*, *t*, *r*) with confidence intervals, effect sizes, degrees of freedom and *P* value noted<br>*Give P values as exact values whenever suitable.* |
| ☒ | ☐ | For Bayesian analysis, information on the choice of priors and Markov chain Monte Carlo settings |
| ☒ | ☐ | For hierarchical and complex designs, identification of the appropriate level for tests and full reporting of outcomes |
| ☒ | ☐ | Estimates of effect sizes (e.g. Cohen's *d*, Pearson's *r*), indicating how they were calculated |

*Our web collection on statistics for biologists contains articles on many of the points above.*

## Software and code

Policy information about availability of computer code

| | |
|---|---|
| Data collection | SPring-8 BL32XU, FlexStation3 (Molecular Devices), My iQ2 (BIORAD), AccuFLEX-γ 8010 (Hitachi), AccuFLEX LCS-8000 liquid scintillation, counter (Hitachi), Guava EasyCyte Plus Flow Cytometer (Merck Millipore), infinite M200 PRO (TECAN) |
| Data analysis | SHIKA, KAMO, XDS, XSCALE, PHASER, SMILES, COOT, PHENIX, Cuemol, GraphPad Prism |

For manuscripts utilizing custom algorithms or software that are central to the research but not yet described in published literature, software must be made available to editors/reviewers. We strongly encourage code deposition in a community repository (e.g. GitHub). See the Nature Research guidelines for submitting code & software for further information.

## Data

Policy information about availability of data

All manuscripts must include a data availability statement. This statement should provide the following information, where applicable:
- Accession codes, unique identifiers, or web links for publicly available datasets
- A list of figures that have associated raw data
- A description of any restrictions on data availability

The structural data have been deposited in the Protein Data Bank under accession number 6KO5 for GHSR-Fab complex and 6KS2 for Fab 7881, respectively.

# Field-specific reporting

Please select the one below that is the best fit for your research. If you are not sure, read the appropriate sections before making your selection.

☒ Life sciences ☐ Behavioural & social sciences ☐ Ecological, evolutionary & environmental sciences

# Life sciences study design

All studies must disclose on these points even when the disclosure is negative.

| | |
|---|---|
| Sample size | The sample size was chosen based on common practice in the field. |
| Data exclusions | The outliers on the luciferase assay were detected and removed based on the Smirnov–Grubbs' test. No exclusion of the data was performed in other experiments. |
| Replication | Experimental findings were reliably reproduced. |
| Randomization | Randomization was not required. |
| Blinding | Blinding was not performed. |

# Reporting for specific materials, systems and methods

We require information from authors about some types of materials, experimental systems and methods used in many studies. Here, indicate whether each material, system or method listed is relevant to your study. If you are not sure if a list item applies to your research, read the appropriate section before selecting a response.

## Materials & experimental systems

| n/a | Involved in the study | |
|---|---|---|
| ☐ | ☒ | Antibodies |
| ☐ | ☒ | Eukaryotic cell lines |
| ☐ | ☐ | Palaeontology |
| ☐ | ☒ | Animals and other organisms |
| ☐ | ☐ | Human research participants |
| ☐ | ☐ | Clinical data |

## Methods

| n/a | Involved in the study | |
|---|---|---|
| ☐ | ☐ | ChIP-seq |
| ☐ | ☒ | Flow cytometry |
| ☐ | ☐ | MRI-based neuroimaging |

## Antibodies

| | |
|---|---|
| Antibodies used | Fab 7881 |
| Validation | The Fab fragment antibody (Fab 7881) specific for the ghrelin receptor is our original as described in the manuscript. |

## Eukaryotic cell lines

Policy information about cell lines

| | |
|---|---|
| Cell line source(s) | Cells used in receptor expression and assays were obtained from Thermo Fisher Scientific (Sf9) and ATCC (HEK293 and CHO). |
| Authentication | No authentication required. |
| Mycoplasma contamination | Cell lines were tested and are free from mycoplasma contamination. |
| Commonly misidentified lines (See ICLAC register) | Cells are not listed in the database. |

## Palaeontology

| | |
|---|---|
| Specimen provenance | Not applicable |
| Specimen deposition | Not applicable |
| Dating methods | Not applicable |

☐ Tick this box to confirm that the raw and calibrated dates are available in the paper or in Supplementary Information.

# Animals and other organisms

Policy information about studies involving animals; ARRIVE guidelines recommended for reporting animal research

| | |
|---|---|
| Laboratory animals | MRL/lpr mice |
| Wild animals | Not applicable |
| Field-collected samples | Not applicable |
| Ethics oversight | All animal experiments described in this study conformed to the guidelines outlined in the Guide for the Care and Use of Laboratory Animals of Japan and were approved by Kyoto University Animal Care Committee (approval No. Med Kyo 16043). |

Note that full information on the approval of the study protocol must also be provided in the manuscript.

# Human research participants

Policy information about studies involving human research participants

| | |
|---|---|
| Population characteristics | Not applicable |
| Recruitment | Not applicable |
| Ethics oversight | Not applicable |

Note that full information on the approval of the study protocol must also be provided in the manuscript.

# Clinical data

Policy information about clinical studies

All manuscripts should comply with the ICMJE guidelines for publication of clinical research and a completed CONSORT checklist must be included with all submissions.

| | |
|---|---|
| Clinical trial registration | Not applicable |
| Study protocol | Not applicable |
| Data collection | Not applicable |
| Outcomes | Not applicable |

# ChIP-seq

## Data deposition

- [ ] Confirm that both raw and final processed data have been deposited in a public database such as GEO.
- [ ] Confirm that you have deposited or provided access to graph files (e.g. BED files) for the called peaks.

| | |
|---|---|
| Data access links<br>*May remain private before publication.* | Not applicable |
| Files in database submission | Not applicable |
| Genome browser session<br>(e.g. UCSC) | Not applicable |

## Methodology

| | |
|---|---|
| Replicates | Not applicable |
| Sequencing depth | Not applicable |
| Antibodies | Not applicable |
| Peak calling parameters | Not applicable |
| Data quality | Not applicable |
| Software | Not applicable |

# Flow Cytometry

## Plots

Confirm that:

- [ ] The axis labels state the marker and fluorochrome used (e.g. CD4-FITC).
- [ ] The axis scales are clearly visible. Include numbers along axes only for bottom left plot of group (a 'group' is an analysis of identical markers).
- [ ] All plots are contour plots with outliers or pseudocolor plots.
- [ ] A numerical value for number of cells or percentage (with statistics) is provided.

## Methodology

| | |
|---|---|
| Sample preparation | Not applicable |
| Instrument | Not applicable |
| Software | Not applicable |
| Cell population abundance | Not applicable |
| Gating strategy | Not applicable |

- [ ] Tick this box to confirm that a figure exemplifying the gating strategy is provided in the Supplementary Information.

# Magnetic resonance imaging

## Experimental design

| | |
|---|---|
| Design type | Not applicable |
| Design specifications | Not applicable |
| Behavioral performance measures | Not applicable |

## Acquisition

| | |
|---|---|
| Imaging type(s) | Not applicable |
| Field strength | Not applicable |
| Sequence & imaging parameters | Not applicable |
| Area of acquisition | Not applicable |

Diffusion MRI ☐ Used ☒ Not used

## Preprocessing

| | |
|---|---|
| Preprocessing software | Not applicable |
| Normalization | Not applicable |
| Normalization template | Not applicable |
| Noise and artifact removal | Not applicable |
| Volume censoring | Not applicable |

## Statistical modeling & inference

| | |
|---|---|
| Model type and settings | Not applicable |
| Effect(s) tested | Not applicable |

Specify type of analysis: ☐ Whole brain ☐ ROI-based ☐ Both

| | |
|---|---|
| Statistic type for inference (See Eklund et al. 2016) | Not applicable |
| Correction | Not applicable |

## Models & analysis

| n/a | Involved in the study |
|---|---|
| ☒ | ☐ Functional and/or effective connectivity |
| ☒ | ☐ Graph analysis |
| ☒ | ☐ Multivariate modeling or predictive analysis |
