# Supplementary material for: Structure of an antagonist-bound ghrelin receptor reveals possible ghrelin recognition mode
Source: Nat Commun. 2020 Aug 19;11:4160. doi: 10.1038/s41467-020-17554-1 (PMC7438500; doi:10.1038/s41467-020-17554-1)
Supplement: Supplementary file 2 — Peer Review File [file 41467_2020_17554_MOESM2_ESM.docx]

Peer Review Information

**Journal:** Nature Communications

**Manuscript title:** Structure of an antagonist-bond ghrelin receptor reveals possible ghrelin recognition mode

**Corresponding author name(s):** Dr Masayasu Kojima

**Editorial notes:**

**none**

**Reviewer comments & decisions:**

**Reviewer comments, first version:**

Reviewer #1 (Remarks to the Author):


The manuscript by Shiimura et. al. describes the crystal structure of the human ghrelin receptor bound to an antagonist. To facilitate crystallogenesis, the authors have modified the receptor sequence and developed a Fab fragment antibody which binds its third intracellular loop. The authors first discussed the antagonist compound 21 binding mode and the architecture of its binding domain. Then, based on a mutagenesis study and functional assays, the authors propose a possible ghrelin binding mode in the Cpd21 binding pocket.
To my knowledge, this study provides the very first high-resolution structure of this clinically relevant GPCR target. I thus believe it is of immediate interest to many people in the GPCR field. Overall the methodology and data are robust and I did not find any flaws that should prohibit its publication.

However I have several comments and questions:

It would be useful for readers to briefly present Compound 21 discovery and pharmacological properties. Is it a neutral antagonist or inverse agonist? This also raises the question of Ghrelin receptor constitutive activity which was previously characterized as significantly high. Could the author comment on this point? In particular in the functional assays presented, is it technically possible to measure the receptor constitutive activity and to assess the effect of the mutations on this activity?
To me it was not clear how the Calcium data were analyzed and compared in particular in the untreated samples (ie -10 on the figures) and between WT and the mutants. Did the authors check the expression level of each constructs?
It would also be interesting to highlight the position of A204 as this is a critical aminoacid responsible in part of the high level of receptor constitutive activity. As the author may know, A204E leads to a loss of receptor constitutive activity and was associated with human short stature (J Clin Invest. 2006;116(3):760-768).
To me, the title is too strong as the mutagenesis and functional data cannot alone provide the structural basis of the Ghrelin binding to GHSR in particular in the absence of computational studies.
"Structure of an antagonist-bound ghrelin receptor reveals possible ghrelin recognition mode." would sound more reasonable.
Also I believe, it is important for the author to mention/discuss the fact that NMR and computational studies recently highlighted that the ghrelin acyl chain is essential to facilitate access of ghrelin to the receptor ligand-binding pocket (PNAS Ferre 2019).


Reviewer #2 (Remarks to the Author):

Du et al. present the first high-resolution structure of the ghrelin receptor (GSHR), a G protein-coupled receptor that has received tremendous interest as a therapeutic target due to its role in appetite regulation, bound to an antagonist. The endogenous agonist of GSHR is a lipidated peptide. Notably, while GSHR is most closely related to peptide-binding GPCRs, the receptor ligand-binding has features reminiscent of lipid-binding GPCRs. Most striking is a feature the authors call the “crevasse”—a gap in between two which seems to be a unifying feature of lipid-binding GPCRs, and distinct from more closely related peptide receptors. The pocket is also “bifurcated” into two regions that are hypothesized to accommodate the peptide chain and the acyl moiety of ghrelin, respectively. The authors use mutagenesis to support the binding mode of the crystallized antagonist and the proposed binding mode of ghrelin.

The manuscript will be of considerable interest to the field due to the therapeutic relevance of the GSHR. Comments and suggestions are listed below.

1. Could the authors please explain their rationale for cutting the resolution at CC1/2 of 62%, and the discrepancy between the completeness reported by the authors vs EDS in the PDB validation report? If the highest resolution bin is 3.3-3.4A, why is the resolution not consistently reported as 3.3A but rather at 3.4A at several places?

2. Please specify what type of omit map (composite, simulated annealing) is shown for Extended Data Fig. 2e.

3. Given that this is the first description of the crystallization construct, it would be appropriate to validate the construct’s function (e.g., radioligand binding with [125I]-ghrelin and/or [3H]-Compound 21), or to cite any relevant literature on whether or not truncation of the N-terminus or thermostabilizing mutations impacts ligand binding. Given the role that the N-terminus plays in ligand binding for many peptide GPCRs, along with the relatively small size of Compound 21 compared to the 28aa endogenous ligand, the modifications may well affect ghrelin binding.

4. The authors allude to aspects of their ELISA screening protocol as “novel” in the methods, but the procedural details are quite sparse and should be expanded. It would also be appropriate to provide a couple of sentences about the antibody selection in the main text if the authors think it is original enough to be a useful strategy for others.

5. The statement in line 63 “Fab 7881 binds to the intracellular loop 3 (ICL3), which is the coupling site of the G protein” is very misleading, since it is hardly the only point of interaction between GPCRs and G proteins.

6. There is some ambiguity in the main text and Extended Data Fig. 2b as to whether Compound 21 and Fab 7881 exhibit positive cooperativity that would indicate they each stabilize similar conformations. The phrasing in the legend of Extended Data Figure 2b is slightly ambiguous whether both curves are in the presence of Compound 21, or if Compound 21 is only present in the Fab complex sample. The main text lines 61-62 makes it sound as though the comparison is apo vs Fab + Compound 21, but line 65 also implies that the Fab has a thermostabilizing effect by itself. Please either clarify the text or add supporting data if appropriate (e.g., thermostability data with Fab and Compound 21 alone, or radioligand binding +/- Fab).

7. Methods for the thermostability experiment (Extended Data Fig. 2b) were omitted from the Materials and Methods section.

8. Given the importance the authors assign to the E1243.33-R2836.55 salt bridge that bifurcates the ligand-binding pocket, it would be helpful to explicitly show this interaction (preferably showing the electron density for these side chains) instead of only as a cartoon in Fig. 2b.

9. The authors may want to consider using a representation similar to Extended Data Fig. 4a in main text Fig. 2c. I think the extended data figure depicts the “gap” much more effectively.

10. The authors discuss the importance of both the “crevasse” and “cavity I” for binding lipid moieties, but they do not ever show clearly how these regions are arranged relative to each other, which might be somewhat confusing to readers who are not comfortable exploring the structural model themselves. They may want to consider adding a figure panel that more clearly depicts the overall topology of the orthosteric site.

11. The calcium signaling data presented in Figure 4 can be affected not only by changes in ligand binding affinity but also by other aspects of receptor expression and function. If possible, either data showing that the expression levels of these mutants are comparable to wild-type or a direct assessment of ghrelin affinity by radioligand binding (as in Extended Data Fig 7) would support the author’s conclusions about the impact of these positions on ligand binding. If this is not possible, the relevant text should be qualified to acknowledge the pleiotropic effects on receptor function that this assay will detect.

12. The contrast in some figure panels is poor and will be difficult to read at a typical print size (e.g., Fig 1- small yellow ligand against orange receptor, Fig 5- red text against red background).

**Author rebuttal, first version:**

**To Reviewer #1**:

(Reviewer's comments)

The manuscript by Shiimura et. al. describes the crystal structure of the human ghrelin receptor bound to an antagonist. To facilitate crystallogenesis, the authors have modified the receptor sequence and developed a Fab fragment antibody which binds its third intracellular loop. The authors first discussed the antagonist compound 21 binding mode and the architecture of its binding domain. Then, based on a mutagenesis study and functional assays, the authors propose a possible ghrelin binding mode in the Cpd21 binding pocket. 
To my knowledge, this study provides the very first high-resolution structure of this clinically relevant GPCR target. I thus believe it is of immediate interest to many people in the GPCR field. Overall the methodology and data are robust and I did not find any flaws that should prohibit its publication.

However I have several comments and questions:

It would be useful for readers to briefly present Compound 21 discovery and pharmacological properties. Is it a neutral antagonist or inverse agonist? This also raises the question of Ghrelin receptor constitutive activity which was previously characterized as significantly high. Could the author comment on this point? In particular in the functional assays presented, is it technically possible to measure the receptor constitutive activity and to assess the effect of the mutations on this activity?
To me it was not clear how the Calcium data were analyzed and compared in particular in the untreated samples (ie -10 on the figures) and between WT and the mutants. Did the authors check the expression level of each constructs? 
It would also be interesting to highlight the position of A204 as this is a critical aminoacid responsible in part of the high level of receptor constitutive activity. As the author may know, A204E leads to a loss of receptor constitutive activity and was associated with human short stature (J Clin Invest. 2006;116(3):760-768).
To me, the title is too strong as the mutagenesis and functional data cannot alone provide the structural basis of the Ghrelin binding to GHSR in particular in the absence of computational studies. 
"Structure of an antagonist-bound ghrelin receptor reveals possible ghrelin recognition mode." would sound more reasonable.
Also I believe, it is important for the author to mention/discuss the fact that NMR and computational studies recently highlighted that the ghrelin acyl chain is essential to facilitate access of ghrelin to the receptor ligand-binding pocket (PNAS Ferre 2019).

(To clear the points that we changed in the revised manuscript, the reviewer's comments are presented again by red letters in the following parts.)

1. It would be useful for readers to briefly present Compound 21 discovery and pharmacological properties.

As suggested by the reviewer, we added a sentence about the discovery of Compound 21 (lines 66–67). Reference #21 contains the synthetic methods and detailed molecular properties.

2. Is it a neutral antagonist or inverse agonist?

In the original article about Compound 21 (reference #21), it does not specify whether Compound 21 is a neutral antagonist or inverse agonist. We then examined the constitutive activity of Compound 21 on ghrelin receptor and found that Compound 21 had no effect on constitutive activity. Hence, we concluded that Compound 21 is a neutral antagonist. We present the data below; however, we have not inserted it into the manuscript because it is mainly concerned with the properties of Compound 21 and is not closely related with the aim of this study. We added the following sentence (lines 67–69): “Compound 21 had no effect on the constitutive activity of the ghrelin receptor, indicating that it is a neutral antagonist (data not shown).”.

3. This also raises the question of Ghrelin receptor constitutive activity which was previously characterized as significantly high. Could the author comment on this point?

As suggested by the reviewer, the high constitutive activity is an important feature of the ghrelin receptor. Then, we have added the following sentences (lines 47–52) with three references (#17–19) and changed the subsequent sentence.

In addition, several reports indicated that the ghrelin receptor exhibits strong ligand-independent activity. The structural basis for its high constitutive activity is thought to be an aromatic amino acid cluster on transmembrane helixes 6 and 7. However, because of the lack of structural information about the ghrelin receptor, the precise mechanisms of its high constitutive activity and activation by acyl-modified ghrelin remained to be elucidated.

4. In particular in the functional assays presented, is it technically possible to measure the receptor constitutive activity and to assess the effect of the mutations on this activity?

To assess the effect of amino acid mutations on constitutive receptor activity, we measured the basal activities of the WT and mutant ghrelin receptors using the NFAT-pGL luciferase reporter vector. The results are presented in Extended Data Figure 8k and 9g. Comments are inserted in lines 122–125, 153–156, and 167-169. In addition, the detailed methods (luciferase assay and statistical analysis) have been included in the Materials and Methods section.

5. To me it was not clear how the Calcium data were analyzed and compared in particular in the untreated samples (ie -10 on the figures) and between WT and the mutants. Did the authors check the expression level of each constructs?

We have added Extended Data Figures 8 and 9 to show the cell surface expression levels of the WT and mutant ghrelin receptors to judge the proper function of the mutant receptors by intracellular Ca^2+^ concentration assay. The following sentences have been added to the text:

Lines 139–140: Note that the cell-surface expression of these mutants was retained (Extended Data Fig. 8d, e).

Lines 155–156: although the cell-surface expression of the mutant receptors was retained at 78.9−112.1% of the WT level.

6. It would also be interesting to highlight the position of A204 as this is a critical aminoacid responsible in part of the high level of receptor constitutive activity. As the author may know, A204E leads to a loss of receptor constitutive activity and was associated with human short stature (J Clin Invest. 2006;116(3):760-768).

We assayed intracellular Ca^2+^ concentration and basal activity of the A204E mutant. We detected severe reductions of mutant receptor activities in both assays (Figures are in next page). These results are similar to those reported in previous papers. We added the following sentence (lines 88–92), as well as reference #24.

Note that the A204E mutation in ECL2 causes loss of constitutive activity of the ghrelin receptor and is associated with familial short stature. In fact, we observed loss of basal receptor activity and a decrease in ghrelin-induced receptor function (data not shown). Thus, mutation of A204 may block ghrelin’s access to the cavities.

7. To me, the title is too strong as the mutagenesis and functional data cannot alone provide the structural basis of the Ghrelin binding to GHSR in particular in the absence of computational studies.

"Structure of an antagonist-bound ghrelin receptor reveals possible ghrelin recognition mode." would sound more reasonable.As suggested by the reviewer, we changed the title to “Structure of an antagonist-bound ghrelin receptor reveals possible ghrelin recognition mode”.

8. Also I believe, it is important for the author to mention/discuss the fact that NMR and computational studies recently highlighted that the ghrelin acyl chain is essential to facilitate access of ghrelin to the receptor ligand-binding pocket (PNAS Ferre 2019).

As suggested by the reviewer, we added the following sentence (lines 164–166). We included the original paper (reference #38) in the references.

A recent study combining NMR with molecular modeling supports the concept that the n-octanoyl moiety of ghrelin is essential for access to the ligand-binding pocket.

**To Reviewer #2:**

(Reviewer's comments)

Du et al. present the first high-resolution structure of the ghrelin receptor (GSHR), a G protein-coupled receptor that has received tremendous interest as a therapeutic target due to its role in appetite regulation, bound to an antagonist. The endogenous agonist of GSHR is a lipidated peptide. Notably, while GSHR is most closely related to peptide-binding GPCRs, the receptor ligand-binding has features reminiscent of lipid-binding GPCRs. Most striking is a feature the authors call the “crevasse”—a gap in between two which seems to be a unifying feature of lipid-binding GPCRs, and distinct from more closely related peptide receptors. The pocket is also “bifurcated” into two regions that are hypothesized to accommodate the peptide chain and the acyl moiety of ghrelin, respectively. The authors use mutagenesis to support the binding mode of the crystallized antagonist and the proposed binding mode of ghrelin.  

The manuscript will be of considerable interest to the field due to the therapeutic relevance of the GSHR. Comments and suggestions are listed below.

1. Could the authors please explain their rationale for cutting the resolution at CC1/2 of 62%, and the discrepancy between the completeness reported by the authors vs EDS in the PDB validation report? If the highest resolution bin is 3.3-3.4A, why is the resolution not consistently reported as 3.3A but rather at 3.4A at several places?

2. Please specify what type of omit map (composite, simulated annealing) is shown for Extended Data Fig. 2e.

3. Given that this is the first description of the crystallization construct, it would be appropriate to validate the construct’s function (e.g., radioligand binding with [125I]-ghrelin and/or [3H]-Compound 21), or to cite any relevant literature on whether or not truncation of the N-terminus or thermostabilizing mutations impacts ligand binding. Given the role that the N-terminus plays in ligand binding for many peptide GPCRs, along with the relatively small size of Compound 21 compared to the 28aa endogenous ligand, the modifications may well affect ghrelin binding.

4. The authors allude to aspects of their ELISA screening protocol as “novel” in the methods, but the procedural details are quite sparse and should be expanded. It would also be appropriate to provide a couple of sentences about the antibody selection in the main text if the authors think it is original enough to be a useful strategy for others.  

5. The statement in line 63 “Fab 7881 binds to the intracellular loop 3 (ICL3), which is the coupling site of the G protein” is very misleading, since it is hardly the only point of interaction between GPCRs and G proteins.

6. There is some ambiguity in the main text and Extended Data Fig. 2b as to whether Compound 21 and Fab 7881 exhibit positive cooperativity that would indicate they each stabilize similar conformations. The phrasing in the legend of Extended Data Figure 2b is slightly ambiguous whether both curves are in the presence of Compound 21, or if Compound 21 is only present in the Fab complex sample. The main text lines 61-62 makes it sound as though the comparison is apo vs Fab + Compound 21, but line 65 also implies that the Fab has a thermostabilizing effect by itself. Please either clarify the text or add supporting data if appropriate (e.g., thermostability data with Fab and Compound 21 alone, or radioligand binding +/- Fab).

7. Methods for the thermostability experiment (Extended Data Fig. 2b) were omitted from the Materials and Methods section.

8. Given the importance the authors assign to the E1243.33-R2836.55 salt bridge that bifurcates the ligand-binding pocket, it would be helpful to explicitly show this interaction (preferably showing the electron density for these side chains) instead of only as a cartoon in Fig. 2b.

9. The authors may want to consider using a representation similar to Extended Data Fig. 4a in main text Fig. 2c. I think the extended data figure depicts the “gap” much more effectively.

10. The authors discuss the importance of both the “crevasse” and “cavity I” for binding lipid moieties, but they do not ever show clearly how these regions are arranged relative to each other, which might be somewhat confusing to readers who are not comfortable exploring the structural model themselves. They may want to consider adding a figure panel that more clearly depicts the overall topology of the orthosteric site.

11. The calcium signaling data presented in Figure 4 can be affected not only by changes in ligand binding affinity but also by other aspects of receptor expression and function. If possible, either data showing that the expression levels of these mutants are comparable to wild-type or a direct assessment of ghrelin affinity by radioligand binding (as in Extended Data Fig 7) would support the author’s conclusions about the impact of these positions on ligand binding. If this is not possible, the relevant text should be qualified to acknowledge the pleiotropic effects on receptor function that this assay will detect.

12. The contrast in some figure panels is poor and will be difficult to read at a typical print size (e.g., Fig 1- small yellow ligand against orange receptor, Fig 5- red text against red background).

(To clear the points that we changed in the revised manuscript, the reviewer's comments are presented again by red letters in the following parts.)

1. Could the authors please explain their rationale for cutting the resolution at CC1/2 of 62%, and the discrepancy between the completeness reported by the authors vs EDS in the PDB validation report? If the highest resolution bin is 3.3-3.4A, why is the resolution not consistently reported as 3.3A but rather at 3.4A at several places?

When merging many datasets into final dataset, as often happens, <I/sig(I)> and CC(1/2) improve as more datasets are added. On the other hand, statistics such as Rsymm and Rmeas tend to exhibit the opposite behavior. For this dataset as well, the resolution limit improved following the merger of a larger number of data sets. When using the scaling program XSCALE, included in the XDS package, even if CC(1/2) is normal, the shell of <I/sig(I)> is not computed and the statistics are not known if Rsymm etc. becomes abnormal. Because we don't know the details of XSCALE's behavior, we tried to obtain "meaningful results" by performing the following calculations. A very low-resolution dataset contributed to the merge, which could cause Rmeas to become abnormal, resulting in <I/sig(I)> not being computed. So we first scaled with all the datasets and selected the datasets that had a better resolution than the specified resolution threshold. This means that datasets with lower resolution than the threshold were rejected for merging. After this selection, we merged the selected datasets and checked the XSCALE results for some resolution-limit thresholds. Consequently, when merged with a resolution threshold of more than 3.5 Å, <I/sig(I)> appears in the output of XSCALE, which was adopted as the final data. As a result of this selection, the CC of the outermost shell (1/2) was 62%.

In addition, the highest resolution is correctly 3.3 Å. It was our mistake. We corrected the highest resolution as 3.3 Å in the text (line 73).

2. Please specify what type of omit map (composite, simulated annealing) is shown for Extended Data Fig. 2e.

The omitted mFo-DFc map of Compound 21 in Extended Data Figure 3f was calculated using the Polder maps in the PHENIX suite. We added reference #54.

3. Given that this is the first description of the crystallization construct, it would be appropriate to validate the construct’s function (e.g., radioligand binding with [125I]-ghrelin and/or [3H]-Compound 21), or to cite any relevant literature on whether or not truncation of the N-terminus or thermostabilizing mutations impacts ligand binding. Given the role that the N-terminus plays in ligand binding for many peptide GPCRs, along with the relatively small size of Compound 21 compared to the 28aa endogenous ligand, the modifications may well affect ghrelin binding.

To validate the construct’s function, we added Extended Data Figure 3c to show specific binding of [^3^H]-Compound 21 to the ghrelin receptor construct. We also added Extended Data Figure 2 to reveal the functions of the N-terminus and found that N-terminal deletion resulted in loss of cell surface expression and receptor activity. We added the following sentence to lines 58–61.

Deletion of the N-terminal 28 amino acids resulted in loss of cell-surface expression and receptor activity, whereas both expression and activity were retained when the C-terminal 20-amino acids were deleted (Extended Data Figure 2a-d).

4. The authors allude to aspects of their ELISA screening protocol as “novel” in the methods, but the procedural details are quite sparse and should be expanded. It would also be appropriate to provide a couple of sentences about the antibody selection in the main text if the authors think it is original enough to be a useful strategy for others.

As suggested by the reviewer, we removed the word “novel” from the Methods section, as we created the original method and published the detailed procedure in 2014. We added reference #45 by Suharni et al.

5. The statement in line 63 “Fab 7881 binds to the intracellular loop 3 (ICL3), which is the coupling site of the G protein” is very misleading, since it is hardly the only point of interaction between GPCRs and G proteins.

As suggested by the reviewer, the statement in line 63 of the original manuscript, “which is the coupling site of the G protein,” is misleading. We removed the sentence.

6. There is some ambiguity in the main text and Extended Data Fig. 2b as to whether Compound 21 and Fab 7881 exhibit positive cooperativity that would indicate they each stabilize similar conformations. The phrasing in the legend of Extended Data Figure 2b is slightly ambiguous whether both curves are in the presence of Compound 21, or if Compound 21 is only present in the Fab complex sample. The main text lines 61-62 makes it sound as though the comparison is apo vs Fab + Compound 21, but line 65 also implies that the Fab has a thermostabilizing effect by itself. Please either clarify the text or add supporting data if appropriate (e.g., thermostability data with Fab and Compound 21 alone, or radioligand binding +/- Fab).

In the thermal stability analysis, Compound 21 is presented in both samples (bRIL–hGHSR–Fab 7881 complex and bRIL–hGHSR). However, we could not obtain ghrelin receptor crystals by adding only Fab 7881 or Compound 21. Both addition of Fab 7881 and Compound 21 are necessary for crystallization. To clarify the sentences describing the thermal stability experiments, we changed the text (lines 64–66) and figure legend of Extended Data Figure 3b (Extended Data Figure 2b in the original manuscript). The changed text is below.

Lines 64–66: Moreover, both a Fab fragment antibody (Fab 7881) specific for the ghrelin receptor and an antagonist, Compound 21, were added to bRIL-conjugated truncated ghrelin receptor to increase thermostability.

Figure legend of Extended Data Figure 3b (Extended Data Figure 2b in the original manuscript):

b, Thermal stability analysis of bRIL–hGHSR–Fab 7881 complex (green) and bRIL–hGHSR (red). Compound 21 is presented in both samples. Fab 7881 increased Tm by 5˚C.

Furthermore, line 60 in the original manuscript (“were added during purification”) might have led to misunderstanding by readers, because it seems that a Fab antibody and Compound 21 were added during all steps of purification. In fact, the Fab antibody and Compound 21 were added during complex formation and the final purification steps. To clarify, we removed “during purification”.

7. Methods for the thermostability experiment (Extended Data Fig. 2b) were omitted from the Materials and Methods section.

Methods for the thermostability experiment have been added in the Materials and Methods section with a reference #44.

8. Given the importance the authors assign to the E1243.33-R2836.55 salt bridge that bifurcates the ligand-binding pocket, it would be helpful to explicitly show this interaction (preferably showing the electron density for these side chains) instead of only as a cartoon in Fig. 2b.

As suggested by the reviewer, we added the electron density 2mFo-DFc map of E124–R283 to Extended Data Figure 4.

9. The authors may want to consider using a representation similar to Extended Data Fig. 4a in main text Fig. 2c. I think the extended data figure depicts the “gap” much more effectively.

As suggested by the reviewer, we changed the original Figure 2c to a new one (Figure 2d), which is similar to Extended Data Figure 6a (Extended Data Figure 4a in the original manuscript). We believe that the new figure depicts the gap more effectively.

10. The authors discuss the importance of both the “crevasse” and “cavity I” for binding lipid moieties, but they do not ever show clearly how these regions are arranged relative to each other, which might be somewhat confusing to readers who are not comfortable exploring the structural model themselves. They may want to consider adding a figure panel that more clearly depicts the overall topology of the orthosteric site.

We added Figure 2b to depict clearly the overall topology of the “crevasse” and “cavities”. Figure 2c is the schematic model of Figure 2b. We think this is the best way to show the topology between the crevasse and the cavities.

11. The calcium signaling data presented in Figure 4 can be affected not only by changes in ligand binding affinity but also by other aspects of receptor expression and function. If possible, either data showing that the expression levels of these mutants are comparable to wild-type or a direct assessment of ghrelin affinity by radioligand binding (as in Extended Data Fig 7) would support the author’s conclusions about the impact of these positions on ligand binding. If this is not possible, the relevant text should be qualified to acknowledge the pleiotropic effects on receptor function that this assay will detect.

As suggested by the reviewer, we examined the cell-surface expression of the mutant ghrelin receptors. We added Extended Data Figure 8 and 9 to show the cell-surface expression levels of the WT and the mutant ghrelin receptors in order to judge the proper function of the mutant receptors based on the intracellular Ca^2+^ concentration assay. The following sentences have been added:

Lines 139–140: Note that the cell-surface expression of these mutants was retained (Extended Data Fig. 8d, e).

Line 155–156: although the cell-surface expression of the mutant receptors was retained at 78.9−112.1% of the WT level.

12. The contrast in some figure panels is poor and will be difficult to read at a typical print size (e.g., Fig 1- small yellow ligand against orange receptor, Fig 5- red text against red background).

As indicated by the reviewer, we added spheres to Compound 21 to sharpen the shape in the Figure 1 and changed the font color into black to increase the contrast of the text in Figure 5.

**Reviewer comments, second version:**

Reviewer #1 (Remarks to the Author):

The points raised in my previous round of review have been satisfactorily addressed.


Reviewer #2 (Remarks to the Author):

The authors have addressed all reviewer comments made during the initial submission. Very minor comments are below.

Reviewer 2 response, point 3:
In the main text comment on [3H]-Compound 21 binding to the ghrelin receptor construct, please comment on how the ligand’s affinity for the crystallography construct (i.e., the data presented in Extended Data Figure 3c) compares to its affinity for the wild-type receptor (i.e., the data shown in Extended Data Figure 7a, j)

Reviewer 2 response, point 11 (main text lines 144-145):
Based on the data shown in Extended data Figure 2e, R283A shows a significant loss in expression. It would be more in keeping with the data to qualify the text along the lines of “Note that substantial cell-surface expression of these mutants was retained (Extended Data Fig. 8d, e).”

Extended data figure 3b:
“Compound 21 is presented” should read “Compound 21 is present.”

**Author rebuttal, second version:**

**To Reviewer #1**:

Reviewer #1 (Remarks to the Author):

The points raised in my previous round of review have been satisfactorily addressed.

We appreciate the previous comments by reviewer #1.

**To Reviewer #2:**

Reviewer #2 (Remarks to the Author):

The authors have addressed all reviewer comments made during the initial submission. Very minor comments are below.

Reviewer 2 response, point 3:
In the main text comment on [3H]-Compound 21 binding to the ghrelin receptor construct, please comment on how the ligand’s affinity for the crystallography construct (i.e., the data presented in Extended Data Figure 3c) compares to its affinity for the wild-type receptor (i.e., the data shown in Extended Data Figure 7a, j)

Reviewer 2 response, point 11 (main text lines 144-145):
Based on the data shown in Extended data Figure 2e, R283A shows a significant loss in expression. It would be more in keeping with the data to qualify the text along the lines of “Note that substantial cell-surface expression of these mutants was retained (Extended Data Fig. 8d, e).”

Extended data figure 3b:
“Compound 21 is presented” should read “Compound 21 is present.”

(To clear the points that we changed in the revised manuscript, the reviewer's comments are presented again by red letters in the following parts.)

1. Reviewer 2 response, point 3:
In the main text comment on [3H]-Compound 21 binding to the ghrelin receptor construct, please comment on how the ligand’s affinity for the crystallography construct (i.e., the data presented in Extended Data Figure 3c) compares to its affinity for the wild-type receptor (i.e., the data shown in Extended Data Figure 7a, j)

As suggested by the reviewer, the following sentence has been added in line 67-69. Because the figure and the *Kd* value of the binding affinity for the WT ghrelin receptor are included in Extended Data Figure 7 (a and j), we added *Kd* values in the parentheses to compare affinities between the construct and the WT receptor.

Line 67-69: although the binding affinity of the construct (dissociation constant, *K*d = 17.2 ± 7.1 nM) was lower than that of the WT ghrelin receptor (*K*d = 4.42 ± 0.76 nM)

2. Reviewer 2 response, point 11 (main text lines 144-145):
Based on the data shown in Extended data Figure 2e, R283A shows a significant loss in expression. It would be more in keeping with the data to qualify the text along the lines of “Note that substantial cell-surface expression of these mutants was retained (Extended Data Fig. 8d, e).”

As suggested by the reviewer, we changed the sentence in line 139-140 in the 1st revised manuscript to the following sentence in line 138-140 to clear the loss of the cell-surface expression of R283A mutant. We think that "Extended data Figure 2e" in the reviewer's comment should be "Extended data Figure 8e".

Line 138-140: Note that the cell-surface expression of E124^3.33^A mutant was retained at 111.5% of the WT level, while that of R283^6.55^A mutant was significantly decreased (66.3% of the WT level) (Extended Data Fig. 8a, d, e).

3. Extended data figure 3b:
“Compound 21 is presented” should read “Compound 21 is present.”

As suggested by the reviewer, we changed the figure legend of Extended Data Figure 3b to "Compound 21 is present".

**Reviewer comments, third version:**

**Author rebuttal, third version:**
